# The Psyche Magnetometry Investigation

**DOI:** 10.1007/s11214-023-00965-z

**Published:** 2023-03-28

**Authors:** Benjamin P. Weiss, José M. G. Merayo, Jodie B. Ream, Rona Oran, Peter Brauer, Corey J. Cochrane, Kyle Cloutier, Linda T. Elkins-Tanton, John L. Jørgensen, Clara Maurel, Ryan S. Park, Carol A. Polanskey, Maria de Soria Santacruz-Pich, Carol A. Raymond, Christopher T. Russell, Daniel Wenkert, Mark A. Wieczorek, Maria T. Zuber

**Affiliations:** 1 Department of Earth, Atmospheric and Planetary Sciences, Massachusetts Institute of Technology (MIT), Cambridge, MA USA; 2 DTU Space, Technical University of Denmark (DTU), Copenhagen, Denmark; 3 Jet Propulsion Laboratory (JPL), California Institute of Technology, Pasadena, CA USA; 4 School of Earth and Space Exploration, Arizona State University, Tempe, AZ USA; 5 Department of Earth and Space Sciences, University of California, Los Angeles, Los Angeles, CA USA; 6 Observatoire de la Côte d’Azur, Centre National de la Recherche Scientifique (CNRS), Laboratoire Lagrange, Université Côte d’Azur, Nice, France

**Keywords:** Fluxgate magnetometer, Gradiometry, Paleomagnetism, Magnetosphere, Dynamo, Psyche, Planetary differentiation, Asteroids and planetesimals

## Abstract

The objective of the Psyche Magnetometry Investigation is to test the hypothesis that asteroid (16) Psyche formed from the core of a differentiated planetesimal. To address this, the Psyche Magnetometer will measure the magnetic field around the asteroid to search for evidence of remanent magnetization. Paleomagnetic measurements of meteorites and dynamo theory indicate that a diversity of planetesimals once generated dynamo magnetic fields in their metallic cores. Likewise, the detection of a strong magnetic moment ($>2\times10^{14}~\text{Am}^{2}$) at Psyche would likely indicate that the body once generated a core dynamo, implying that it formed by igneous differentiation. The Psyche Magnetometer consists of two three-axis fluxgate Sensor Units (SUs) mounted 0.7 m apart along a 2.15-m long boom and connected to two Electronics Units (EUs) located within the spacecraft bus. The Magnetometer samples at up to 50 Hz, has a range of $\pm80{,}000~\text{nT}$, and an instrument noise of $39~\text{pT}\,\text{axis}^{-1}\,3\sigma $ integrated over 0.1 to 1 Hz. The two pairs of SUs and EUs provide redundancy and enable gradiometry measurements to suppress noise from flight system magnetic fields. The Magnetometer will be powered on soon after launch and acquire data for the full duration of the mission. The ground data system processes the Magnetometer measurements to obtain an estimate of Psyche’s dipole moment.

## Introduction

### Psyche Mission Overview

The Psyche mission aims to characterize the structure, composition, geology and origin of the main belt asteroid (16) Psyche (Elkins-Tanton et al. 2022; Polanskey et al. 2023). With a mean radius, $R_{\mathrm{P}}$, of $\sim111$-km (Shepard et al. 2021), Psyche is likely the largest iron-rich body in the solar system and will be the first small, metal-rich world to be encountered by a spacecraft. The mission has three science goals: (1) understand a previously-unexplored building block of planet formation: iron-rich planetesimals; (2) look inside terrestrial planets, including Earth, by directly examining the interior of a differentiated body, which otherwise could not be seen and (3) explore a new type of world that is rich in metal rather than in rock or ice. One of the three science instruments is the Magnetometer, which will measure the magnetic field around the asteroid. Here we describe the science objectives of the Magnetometry Investigation, their relationship to mission objectives, the context of previous magnetic studies of asteroids and meteorites, the design, performance, and accommodation of the instrument, and the expected data products and calibration pipeline.

This article is structured as follows. In the rest of Sect. 1, we describe the science objectives of the Magnetometry Investigation, how these translate into high level engineering requirements, and an overview of the Magnetometer instrument. In Sect. 2, we review our understanding of remanent magnetization in meteorites and small bodies. In Sect. 3, we describe the design, ground calibration, accommodation, operations and performance of the Magnetometer. In Sects. 4 and 5, we describe the data and in-flight calibration pipelines. We summarize the Investigation in Sect. 6.

### Science Objectives

The three mission science goals (Sect. 1.1) are addressed with five science objectives (Polanskey et al. 2023). Of these, the Magnetometry Investigation was designed to address the central mission science objective: determine whether Psyche formed from a core or if it is primordial unmelted material (Elkins-Tanton et al. 2022).

Based upon the currently available data, the team has assembled models for what Psyche might be (Elkins-Tanton et al. 2020). The asteroid’s bulk density is estimated to be between 3,400 and $4{,}100~\text{kg}\,\text{m}^{-3}$. Because meteoritic metal has a density of about $7{,}000~\text{kg}\,\text{m}^{-3}$, Psyche must be a mixture of metal and lower-density components including silicates and/or pore space. Given the range of likely secondary components, Psyche is probably between ∼30–60 vol.% (∼70–100 wt.%) metal (Elkins-Tanton et al. 2020; Zhang et al. 2022). Psyche thus appears to be more metal-rich than the Earth (∼32 wt.% metal) (McDonough and Sun 1995) and ordinary (LL, L, and H) chondritic materials (∼2–20 wt.% metal) (Dunn et al. 2010).

Core formation may be the simplest model for forming a metal-enriched parent body for Psyche. Our current fiducial model for the asteroid’s formation is therefore that it is the remains of a differentiated body that had formed a metallic core. Alternatively, Psyche might be an undifferentiated body consisting of a highly reduced, low-FeO material. High density, low-FeO meteorite analog candidates include enstatite, CH, and CB chondrites (Dibb et al. 2022; Elkins-Tanton et al. 2020).

**Fig. 1:**
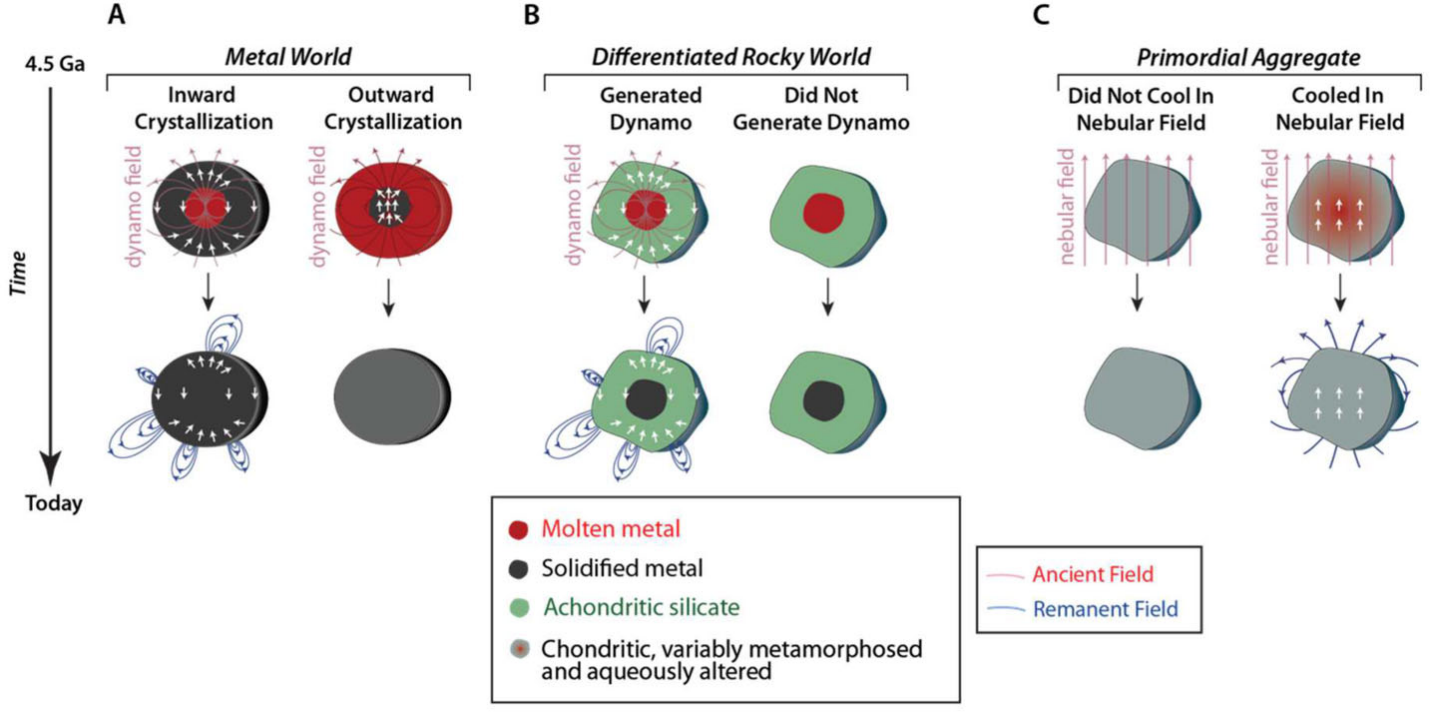
Some possible magnetization states for Psyche and their relationships to possible formation and evolution models. Top and bottom rows show Psyche’s structure 4.5 billion years ago (Ga) and today, respectively. Red and blue fields denote dynamo and remanent magnetic fields. Red, dark grey and green shading denote molten metal, solid metal, and melted silicates, respectively in (**A**) and (**B**). Grey and red-gray shading denote unheated and heated but unmelted primordial materials in (**C**). (**A**) Formation as a differentiated mantle-stripped metallic planetesimal with a molten core. Such a body could crystallize from the outside-in (left) or inside-out (right). Both scenarios might generate a dynamo but only the former could produce remanent magnetization. (**B**) Formation as a differentiated or partially differentiated body with a silicate crust and molten iron core. Such a body might (left) or might not (right) generate a dynamo that could magnetize the silicate mantle. Similarly, a mantle-stripped metallic world (**A**) might also not generate a dynamo (not shown here). (**C)** Formation as an unmelted primordial aggregate. Such a body might not (left) or might (right) have been heated and/or aqueously altered while a nebular magnetic field was present. Only the latter could magnetize the asteroid

Measurement of a strong remanent magnetic field would perhaps be the single most definitive determiner of Psyche’s origin: if Psyche is magnetized, the long-past action of a core dynamo (Sect. 1.3) is the most likely cause (Fig. 1). Psyche would thus be determined to be a core or parts of a core. On the other hand, if Psyche is composed of metal-rich undifferentiated material, it would never have generated a dynamo. The absence of a remanent field would certainly be indeterminate: even if Psyche was a core, its materials may have not been magnetized by an existing dynamo field, or else the core may never have generated a dynamo field.

**Fig. 2:**
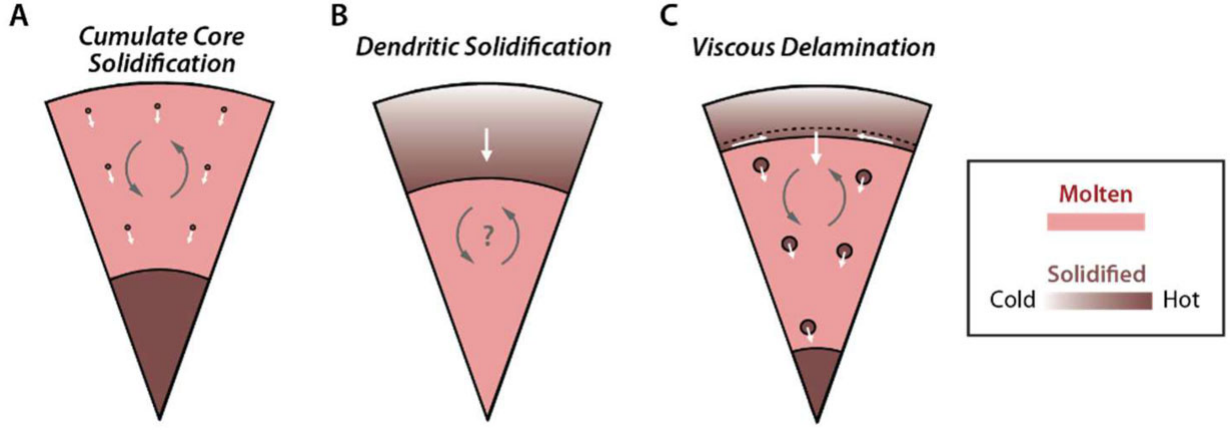
Three evolutionary scenarios for an inwardly crystallizing mantle-stripped metallic planetesimal. ($\mathbf{A}$) The crystallites, which are denser than the surrounding liquid, sink to the bottom and accumulate as a detrital core that remains above the Curie temperature during the lifetime of any convective dynamo (Scheinberg et al. 2015). Such a body would not be magnetized. ($\mathbf{B}$) The core undergoes dendritic inward crystallization without foundering, thereby building a solid crust. However, this crust may only cool below the Curie temperature after any convection ceases, such that it would not be magnetized (Scheinberg et al. 2015). ($\mathbf{C}$) A stable crust forms and undergoes large-scale foundering at its low viscosity, warm base. Such a body might maintain a dynamo sufficiently long for the outer part of the crust to cool below its Curie point and become magnetized (Neufeld et al. 2019). Light red color denotes molten iron and brown shading denotes solid iron with varying temperature

Psyche could become magnetized by the dynamo if part of it cooled and/or underwent low-temperature recrystallization while the field was still being generated in the asteroid’s molten interior (Fig. 1). Acquiring magnetization would require that ferromagnetic materials on Psyche were below their characteristic critical ordering temperatures (e.g., $780~^{\circ}\text{C}$ for the Fe mineral kamacite and as low as $320~^{\circ}\text{C}$ for the FeNi mineral tetrataenite) while the dynamo was still active (Scheinberg et al. 2017). Therefore, such a body could have recorded a paleofield only if it crystallized from the outside-in (rather than from the inside-out) where the materials were sufficiently cool. Inward crystallization will occur if the liquidus (melting temperature) increases more rapidly with pressure than does the internal temperature profile of the body, a condition that is likely but not guaranteed for small bodies depending on particular on their sulfur-contents (Williams 2009). The outer layers could be composed of metal (if Psyche is a mantle-stripped metallic body) (Neufeld et al. 2019; Scheinberg et al. 2015), an achondritic silicate mantle (if Psyche is fully differentiated but its mantle was not fully stripped away) (Bryson et al. 2015; Weiss et al. 2008) or even an undifferentiated, primordial crust (if Psyche is partially differentiated) (Weiss and Elkins-Tanton 2013). However, even for an inwardly crystallized body, only some crystallization scenarios would lead to magnetization acquisition (Fig. 2). Alternatively, a mantle-stripped core may also have solidified from the inside outward such that there was no solid core material lying above the dynamo.

Although an undifferentiated body could not have generated a dynamo, an external field like that of the solar nebula (Weiss et al. 2021) might magnetize such a body if it was aqueously altered and/or thermally metamorphosed before the nebula dispersed (Courville et al. 2022). Such magnetization could potentially be distinguished from an internal field by the fact that it may be unidirectional throughout the body given that the nebular field is expected to be spatially uniform over scales of at least $10^{-2}$ AU (Wang et al. 2017). Furthermore, such a body could be identified by the fact that it is expected to compositionally and mineralogically resemble chondritic materials as indicated by Gamma-Ray and Neutron Spectrometer and Multispectral Imager data (Elkins-Tanton et al. 2022).

### Detecting Psyche’s Magnetic Field

Advecting planetary metallic cores can generate magnetic fields by the dynamo process in which motion of a conductive fluid amplifies a seed magnetic field by the process of induction (Moffatt and Dormy 2019). Such a dynamo field can then imprint natural remanent magnetization (NRM), which is the semi-permanent alignment of electron spins in ferromagnetic minerals (see Table 1 for a list of all acronyms and variables used in this article). As introduced in Sect. 1.2), NRM can take the form of thermoremanent magnetization when ferromagnetic minerals cool through their critical ordering temperatures or as crystallization remanent magnetization when these minerals crystallize or form as transformation products from pre-existing minerals during aqueous alteration or subsolidus cooling (Dunlop and Özdemir 1997). Although planetesimal dynamos are thought to only have lasted for at most several hundred million years (Ma) due to the efficient radiative cooling of small bodies (e.g., Maurel et al. 2021 and Nichols et al. 2021). NRM can potentially persist until today (Shah et al. 2018). The Magnetometry Investigation addresses the key mission science objective by constraining the remanent magnetization of Psyche.

**Table 1:** Acronyms and variables used in this manuscript

Acronym or variable	Definition
MIT	Massachusetts Institute of Technology
DTU	Technical University of Denmark
JPL	Jet Propulsion Laboratory
CNRS	Centre National de la Recherche Scientifique
NRM	natural remanent magnetization
IMF	interplanetary magnetic field
SU, SU1, SU2	Sensor Unit, Sensor Unit 1, Sensor Unit 2
EU, EU1, EU2	Electronics Unit, Electronics Unit 1, Electronics Unit 2
FS	Flight system (frame)
Ma	million years
CAI	calcium aluminum-rich inclusion
XPEEM	X-ray photoemission electron microscopy
MG	main group
VFM	Vector Field Magnetometer
CHAMP	Challenging Microsatellite Project
CDC	Compact Detector Coils
CSC	Compact Spherical Coils
FPGA	field-programmable gate array
eu	engineering units
LVDS	low-voltage differential signaling
UART	universal asynchronous receiver-transmitter
SPUS	SPUS (Swarm Packet Utilization Standard)
PPS	pulse-per-second
STM	spacecraft time message
DSOC	Deep Space Optical Communication
TWTA	traveling-wave tube amplifiers
CCSDS	Consultative Committee for Space Data Systems
CFDP	CCSDS File Delivery Protocol
PBF	Psyche-body-fixed (frame)
PSO	Psyche-solar-orbit (frame)
J2E	Psyche-centered EMO J2000 (frame)
SCLK	spacecraft clock
NAIF	Navigation and Ancillary Information Facility
CDF	Common Data Format
SADA	solar array drive assembly
$R_{\mathrm{P}}$	mean radius of Psyche
$m_{\mathrm{s}}$	specific NRM
$M_{\mathrm{P}}$	dipole moment of Psyche
+*X*,+*Z*,−*Z*	identities of decks of Psyche spacecraft bus
$r_{P}$	Psyche-centric distance
$B_{\mathrm{MP}}$	equatorial remanent magnetic field
$P_{\mathrm{B}}$	magnetic pressure
$\mu _{0}$	permeability of free space
$P_{\mathrm{sw}}$	solar wind dynamic pressure
$\rho _{\mathrm{sw}}$	solar wind density
$v_{\mathrm{sw}}$	solar wind velocity
$m_{\mathrm{H}}$	mass of hydrogen atom
$n_{\mathrm{sw}}$	solar wind number density
$m_{\mathrm{P}}$	magnetization of Psyche
*F*	upstream field enhancement
$r_{\mathrm{MP}}$	radius of Psyche’s magnetosphere
$f_{s}$	sampling frequency of Magnetometer
*x*,*y*,*z*	Cartesian coordinates for measurement location
*r⃗*	position with respect to spacecraft bus
$\vec{r}_{1}$	position of SU1
$\vec{r}_{2}$	position of SU2
$\vec{B}_{\mathrm{M}}$	measured magnetic field
Δ*B⃗*	difference between field measured at SU2 and SU1
$\vec{B}_{\mathrm{amb}}$	ambient magnetic field
$\vec{B}_{\mathrm{FS}}$	flight system magnetic field
$\vec{B}_{\mathrm{FS}, \mathrm{DC}}$	steady part of flight system magnetic field
$\vec{B}_{\mathrm{FS}, \mathrm{AC}}$	time-variable part of flight system magnetic field
$\vec{B}_{\mathrm{N}}$	instrument noise
$\vec{B}_{\mathrm{N},2}$ and $\vec{B}_{\mathrm{N},1}$	instrument noise for measurements acquired with SU2 and SU1
*a*	index of field component measured by SU1
*b*	index of field component measured by SU2
and $\alpha _{ab}$	coupling matrix and its entries for SU1 and SU2
$\vec{B}_{\mathrm{amb}} '$	estimated ambient magnetic field
$\vec{M}_{\mathrm{FS}} '$	estimated magnetic moment of flight system
$\vec{B}_{\mathrm{ppr}, n}$	Partially Processed (ppr) data
$\vec{O}_{n}$	offset vector for SU*n*/EU*n*
$\boldsymbol{S}_{\boldsymbol{n}}$	scale factor matrix for SU*n*/EU*n*
$\boldsymbol{V}_{\boldsymbol{n}}$	orthogonalization matrix for SU*n*
$\boldsymbol{R}_{\boldsymbol{n}}$	rotation matrix for SU*n*/EU*n*
*n*	index for SU1 and SU2
$t_{\mathrm{ABS}}$	spacecraft time of the start of each packet
$t_{m,a}$	measurement time of each sample in a science packet
$t_{\mathrm{MAG}, a}$	time for each science packet measurement relative to the start of the packet
$\Delta _{\mathrm{PPS}}$	offset of the time of a field measurement with respect to the arrival of the PPS
$t_{d}$	instrument latency
*c*	index for each of c50 measurements in a science packet
$\vec{B}_{\mathrm{raw},n}$	Raw data vector for SU*n*
$eu_{n,a}$	entry for *a*^th^ field component in Raw data vector for SU*n*
$T_{n,i}$	temperature reading for SU*n*/EU*n* and associated CSC, CDC, or EU
*i*	index for CSC, CDC, or EU temperature sensors
$C_{n,0,i} \dots C_{n,7,i}$	coefficients for each temperature sensor from ground calibration
$VET_{n,i}$	voltage measured by each temperature sensor
$\vec{E}_{n}$	vector of linearized field data for SU*n*
$E_{n,a}$	entry for *a*^th^ field component in $\vec{E}_{n}$
$k_{n,2,j} $ and $k_{n,3,j}$	linearity parameters from ground calibration
$O_{n,a}$	entry for *a*^th^ field component in $\vec{O}_{n}$
$O_{n,0,a}$, $O_{n, \mathrm{CDC},a}$, $O_{n, \mathrm{EU},a} $ and $O_{n,t,a}$	coefficients for offset matrix from ground calibration
$\Delta O_{n, \mathrm{CDC},a} ( T_{n, \mathrm{CDC}} )$	look-up table for offset matrix from ground calibration
$t_{L}$	time since launch (in years)
$S_{n,a}$	entry for *a*^th^ field component in $\boldsymbol{S}_{\boldsymbol{n}}$
$S_{n,0,a}$, $S_{n, \mathrm{CSC},a}$, $S_{n, \mathrm{EU},a}$, and $S_{n,t,a}$	coefficients for scale factor matrix from ground calibration
$u_{n,1}$, $u_{n,2}$, and $u_{n,3} $	coefficients for orthogonalization matrix from ground calibration
$\vec{B}_{\mathrm{cal},\mathrm{FS}, n}$	Partially Processed data rotated into FS coordinates
*f*	frequency of magnetic field
$\vec{B}_{\mathrm{cal},\mathrm{zl},n}$	Partially Processed data rotated into FS coordinates with zero-level correction
$\vec{B}_{\mathrm{zl}, n}$	Estimate of the Magnetometer zero-level
$\vec{B}_{\mathrm{SA}, n}$	solar array magnetic field
$\theta _{S}$	SADA angle
$\vec{B}_{\mathrm{cal},\mathrm{zl},\mathrm{SA},n}$	Partially Processed data rotated into FS coordinates with zero-level and solar array correction applied
$\vec{B}_{\mathrm{cal},\mathrm{zl},\mathrm{SA},\mathrm{FDF},n}$	Partially Processed data rotated into FS coordinates with zero-level and solar array correction applied and frequency domain filtering applied
$f_{\mathrm{FDF}}$	frequency domain filtering function
$\vec{B}_{\mathrm{cal},\mathrm{zl},\mathrm{SA},\mathrm{FDF},\mathrm{SC}, n}$	Partially Processed data rotated into FS coordinates with zero-level and solar array correction applied, frequency domain filtering applied, and step-changes suppressed
$f_{\mathrm{SC}}$	field step change suppression function
$\vec{B}_{\mathrm{cal}, n}$	Calibrated data
$\boldsymbol{R}_{q}$	rotation matrix from FS to J2E, PBF, or PSO frames
*q*	index for J2E, PBF, or PSO frames
$\vec{B}_{\mathrm{der}, q}$	Derived data
$r_{0}$	reference distance from Psyche
$\theta _{P}$	Psyche-centric latitude
$\phi _{P}$	Psyche-centric longitude
$\vec{B}_{\mathrm{der}, \mathrm{i}} $	part of derived field generated internally to $r_{0}$
$\vec{B}_{\mathrm{der}, \mathrm{e}} $	part of derived field generated externally to $r_{0}$
$\vec{r}_{P}$	position with respect to Psyche
$\vec{B}_{\mathrm{der},\mathrm{i}} '$	estimate of Psyche’s internal field
$\vec{B}_{\mathrm{der},\mathrm{e}} '$	estimate of the external field at Psyche
$g_{l}^{m, \mathrm{i}}$ and $h_{l}^{m, \mathrm{i}}$	internal Gauss coefficients
*l*	degree of Gauss coefficients
*m*	order of Gauss coefficients
$R_{l}$	power spectrum of field
$\vec{B}_{\mathrm{cal},\mathrm{SA},\mathrm{FDF},\mathrm{SC}, n}$	Partially Processed data rotated into FS coordinates with solar array correction applied, frequency domain filtering applied, and step-changes suppressed
$\boldsymbol{A}_{\boldsymbol{n}}$	covariance matrix of magnetic field data used in solar wind monitoring
$\vec{b}_{n}$	Right hand size of system of equations solved for zero-level estimates using solar wind monitoring
$B_{x}$, $B_{y}$, $B_{z} $	Cartesian components of $\vec{B}_{\mathrm{cal},\mathrm{SA},\mathrm{FDF},\mathrm{SC}, n}$

*Notes:* The top of the table lists acronyms in order of appearance and the bottom lists variables in order of appearance. The first column lists acronym or variable and second column lists its definition

Gravity power spectra of planetary bodies have been empirically found to be well-described by the so-called Kaula rule, which assumes that planetary gravity fields reflect randomly distributed density anomalies of small spatial coherence distributed throughout the outer regions of the bodies and where gravitational stress is constant and limited by crustal elastic strength (Ermakov et al. 2018; Zuber et al. 2022). However, there is no equivalent a priori estimate for the intrinsic magnetic field of a magnetized body because magnetization depends on the unknown past magnetic field history experienced by the body and the unknown form of any magnetization (e.g., the various aforementioned forms of NRM as well as induced magnetization). As such, the Magnetometry Investigation was designed to distinguish between a broad range of possible magnetization states that relate to aspects of the body’s, structure origin and geologic history.

The Psyche Magnetometry Investigation is designed to be capable of detecting an asteroid magnetic field that is sufficiently strong to be the product of a past dynamo. The specific NRM intensities, $m_{\mathrm{s}}$, of known iron meteorites have been found to range from $\sim10^{-4}$ to $\sim10^{-2}~\text{Am}^{2}\,\text{kg}^{-1}$, where the latter value excludes samples most likely to have been remagnetized by hand magnets (Pesonen et al. 1993) (see Sect. 2.2). In the absence of a magnetic equivalent for the Kaula rule, we assume as a limiting case that any fine-scale NRM in Psyche formed by a dynamo has an intensity at the lower end of this range. It has been found that the crustal magnetic fields for Earth, Mars, and the Moon are each well described by magnetization models consisting of randomly oriented dipoles, perhaps with some lateral spatial correlation (Voorhies 2008; Voorhies et al. 2002; Wieczorek 2018). As such, we also assume that Psyche’s shell can be described by such a pattern (see also Sect. 4.6.3).

To estimate the thickness of such a magnetized layer, we consider a recent thermal evolution model of a Psyche-like, mantle-stripped iron planetesimal which found that the outer 40% of its radius could be magnetized by an internal core dynamo (Neufeld et al. 2019). If we also therefore assume Psyche’s crust is randomly magnetized with a spatial scale given by $\sim40\%$ of its current radius (e.g., $\sim40$-km coherence scale) and with a fine-scale magnetization at the lower end of known iron meteorites, we find that Psyche would have a dipole moment $M_{\mathrm{P}} = 2\times10^{14}~\text{Am}^{2}$. The sole level 1 requirement of the Magnetometry Investigation is to be able to detect this limiting magnetic moment. Note that if we instead assume a fine-scale magnetization at the upper end of known iron meteorites with 40-km coherence scale, the moment would be two orders of magnitude larger than this value (Oran et al. 2022), while a uniformly magnetized body with this magnetization would be three orders of magnitude larger. Note that although a spherical body containing magnetization acquired solely by a steady field of internal origin will produce no external remanent field (Runcorn 1975), Psyche’s nonsphericity (Shepard et al. 2021), combined with the likelihood of time-variability of the dynamo field and magnetostatic interactions between metallic materials within Psyche mean that any dynamo-generated remanent magnetization in the asteroid will almost certainly generate an external field (e.g., Aharonson et al. 2004; Stephenson 1976; Srnka 1976).

**Fig. 3:**
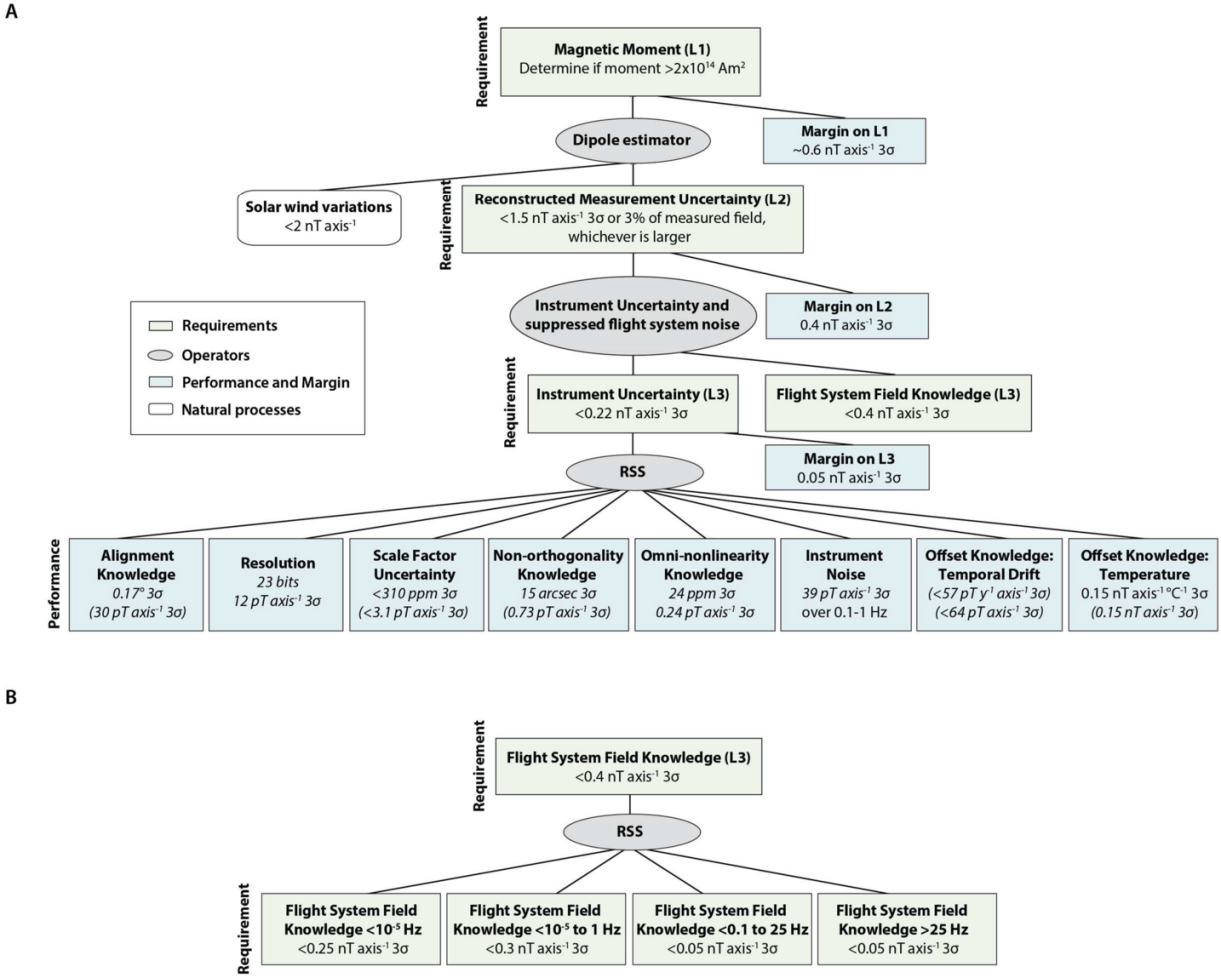
Magnetometer and flight system knowledge requirements, performance, and uncertainty budget. Green boxes contain requirements, diagonal lines denote the flowdown to child requirements and performance, blue boxes contain performance and margin, white box contains uncertainty from solar wind variations and ovals denote operators (RSS = sum in quadrature). (**A**) Overall budget. The level 1 requirement is that the Magnetometry Investigation be able to detect a remanent field from (16) Psyche if the asteroid’s moment exceeds $2\times 10^{14}~\text{Am}^{2}$. The level 2 requirement is that the Reconstructed Measurement Uncertainty, which combines Instrument Uncertainty with uncertainty in Flight System Field Knowledge, enables such a detection given typical solar wind variations in the asteroid belt. The Instrument Uncertainty is composed of uncertainties in knowledge of the orientation of each Sensor Unit (SU) relative to the Flight System (FS) frame (Alignment Knowledge), digitization (Resolution), knowledge of the scale factors, $\boldsymbol{S}_{\boldsymbol{n}}$ (Scale Factor Uncertainty), knowledge of the departure from orthogonality for the SU axes (Non-orthogonality Knowledge), knowledge of the interactions between the three SU axes (Omni-nonlinearity Knowledge), instrument noise integrated over 0.1–1 Hz (Instrument Noise), knowledge of the long-term drift of the offsets, $\vec{O}$ (Offset Knowledge: Temporal Drift), and knowledge of the offset dependence on temperature (Offset Knowledge: Thermal). The requirement levels (L1 = level 1, L2 = level 2, and L3 = level 3) are listed in parentheses next to each requirement name. The requirements assume measurements acquired in a 10 nT ambient field (for the Alignment Knowledge, Resolution, Scale Factor Uncertainty, Non-orthogonality Knowledge, and Omni-nonlinearity Knowledge) with temperature variations at the sensor cores of $\pm1~^{\circ}\text{C}$ (for Offset Knowledge: Temperature), and an assumed lapse of 402 d since the last zero-level field correction (for Offset Knowledge: Temporal Drift; Sect. 4.4). (**B**) Breakdown of total Flight System Field Knowledge requirement. This flows down to requirements on knowledge of the integrated field in four frequency ranges after use of planned mitigations (gradiometry, zero-level corrections, and corrections for solar array fields): $< 10^{-5}$ Hz, $10^{-5}$ to 0.1 Hz, 0.1 to 25 Hz, and 25 Hz to 100 kHz

The Magnetometry Investigation constrains the asteroid’s magnetic moment by sensing its remanent magnetic field. The minimum detectable magnetic moment can be roughly translated into a minimum detectable field as a function of distance from Psyche under the approximation that Psyche’s magnetic field is that of a dipole in a vacuum, such that the field scales as $r_{P}^{-3}$ for Psyche-centric distance, $r_{P}$. The Magnetometer will continuously acquire data after launch including during mapping orbits at increasingly lower altitudes (Polanskey et al. 2023). At the two closest mapping orbits C and D, which are currently estimated to have altitudes of 75 and 190 km (Polanskey et al. 2023), the level-1 minimum moment has a polar field of $\sim6~\text{nT}$ and $\sim2~\text{nT}$. The Magnetometry Investigation is designed such that these remanent fields at these altitudes should be larger than estimated noise sources. The latter are expected to be dominated by fluctuations of the interplanetary magnetic field (IMF) ($\sim \pm 2~\text{nT}$ per axis in the asteroid belt), Instrument Uncertainty (intrinsic instrument noise, estimated to be $<0.17~\text{nT}\,\text{axis}^{-1}\,3\sigma $), and Flight System Field Knowledge (uncertainty in knowledge of the flight system field after it has been suppressed during ground data processing; required to be $<0.4~\text{nT}\,\text{axis}^{-1}\,3\sigma $) (see Sects. 3 and 4). The Magnetometry Investigation’s single level 2 requirement is that the combined Instrument Uncertainty and Flight System Field Knowledge, termed the Reconstructed Measurement Uncertainty, be less than $1.5~\text{nT}\,\text{axis}^{-1}\,3\sigma $ (Fig. 3). We currently estimate that we meet this requirement with a margin of $0.4~\text{nT}\,\text{axis}^{-1}\,3\sigma$.

### Overview of the Psyche Magnetometer

**Fig. 4:**
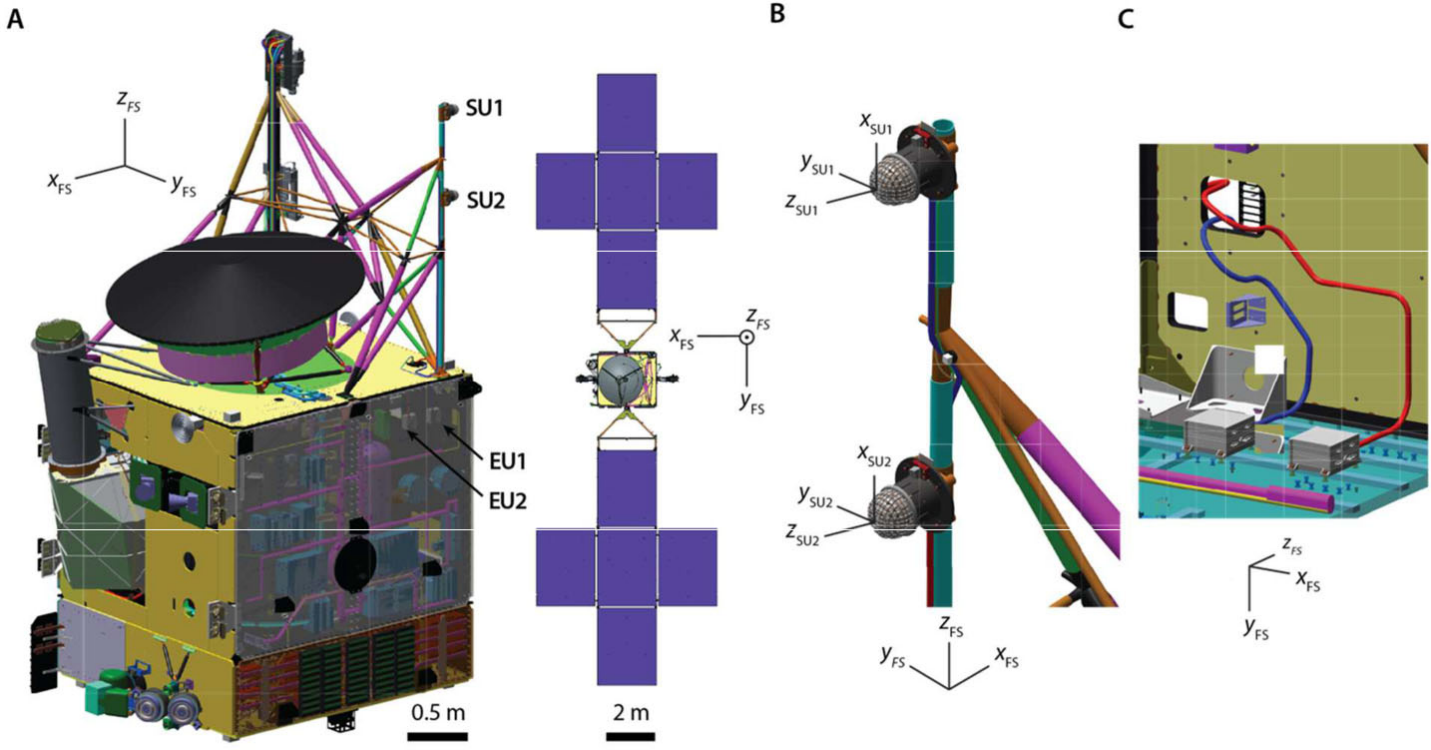
Psyche spacecraft and location of Magnetometer Sensor Units (SUs) and Electronics Units (EUs). ($\mathbf{A}$) SU1 and SU2 are mounted along a boom of length 2.15 m pointing away from the $+Z$ deck. Some of the most magnetic subsystems were placed at distal locations on the spacecraft: Hall thrusters ($-Z$ deck), Deep Space Optical Communication (DSOC) ($+X$ deck). ($\mathbf{B}$) Close-up of SUs. ($\mathbf{C}$) Close-up of EUs and harnesses inside spacecraft bus. Compasses show orientation of FS coordinate system

**Fig. 5:**
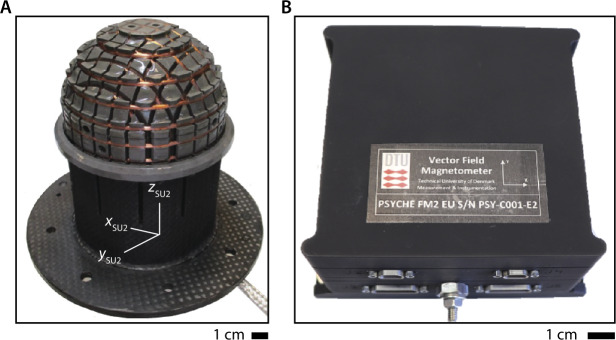
Photographs of Flight Model 2. (**A**) Sensor Unit 2 (SU2). Compass shows orientation of SU2 coordinate system. (**B**) Electronics Unit 2 (EU2)

The Psyche Magnetometer is designed to meet this level 2 requirement with margin. It consists of two three-axis fluxgate sensor units (SUs) on a 2.15-m long boom, each connected by a harness to electronics units (EUs) within the spacecraft bus (Figs. 4, 5). Each EU has two redundant two sides (named A and B) that can connect to the same SU via a cross-strapping board that switches the analog signals. The outboard sensor, located at the outer end of the boom, is named Sensor Unit 1 (SU1) and the inboard sensor, located 0.7 m inward of the end of the boom, is named Sensor Unit 2 (SU2). The two reasons that the mission carries two sensors are to enable gradiometry measurements to reduce noise from the flight system magnetic fields and to provide redundancy. With respect to the latter, the Magnetometry Investigation is designed to meet its level 1 requirement in the event of failure of either SU1 or SU2.

MIT leads the science investigation, operations, ground data processing and trending, DTU designed, built, and calibrated the Magnetometer and leads anomaly resolution, and the Jet Propulsion Laboratory leads commanding. MIT, JPL, and the University of California, Los Angeles jointly developed the level 1 and level 2 Magnetometer science requirements. All four institutions will participate in science operations and data analysis.

## Planetesimal and Asteroid Magnetism

### Introduction to Remanent Magnetization

Most meteorites are samples of planetesimals and many contain NRM records of past magnetic fields on these bodies. The ferromagnetic minerals carrying these records are mainly iron-nickel metals, iron oxides, and iron sulfides. The NRM intensities of meteorites depends on their mineralogy, grain size, history of exposure to ancient fields, and preservation state.

The origin of meteorite NRM was debated for decades following its identification in the 1960s. However, it is now understood that some meteorites acquired their magnetic records during or after their formation during the first several Ma to hundreds of Ma after solar system formation (Weiss et al. 2010). Two main field sources have been identified for known meteorites: a dynamo field generated by advection of a metallic core in the meteorite parent body (Bryson et al. 2019a; Weiss et al. 2008) and a field generated by the solar nebula (Fu et al. 2014; Borlina et al. 2021; Weiss et al. 2021). Such NRM can survive to the present day depending on a meteorite’s ferromagnetic crystal sizes and mineralogy, which control the resistance to remagnetization by thermal metamorphism, shocks, terrestrial weathering and contact with hand magnets (Weiss et al. 2010; Gattacceca et al. 2010; Uehara et al. 2012).

### Magnetic Record of Meteorite Parent Bodies

#### Introduction

NRM attributed to early solar system magnetic fields has been identified in several chondrite and achondrite groups. Here, we focus on recent advances in revealing ancient magnetic records carried by chondrites, iron meteorites and stony-iron meteorites. Paleomagnetic studies conducted on basaltic achondrites, which sample the silicate mantles of differentiated planetesimals, were more recently reviewed elsewhere (Scheinberg et al. 2017).

#### Chondrites

Bulk chondrites and their consitutent chondrules have been found to contain ancient NRM. Individual chondrules, which cooled as free-floating objects in the solar nebula, have been found to contain pre-accretional thermoremanent NRM from cooling in the presence of a nebular magnetic field (Weiss et al. 2021). This field appears to have persisted until about $\sim4$ Ma after the formation of calcium aluminum-rich inclusions (CAIs) (Wang et al. 2017; Weiss et al. 2017; Borlina et al. 2022). Because chondrules accreted onto their parent body in random orientations, such pre-accretional NRM is expected to be randomly oriented within the parent body (Weiss and Elkins-Tanton 2013), as has now been observed for two chondrite groups (Borlina et al. 2021; Fu et al. 2014). Given, the measured chondrule-scale NRM intensities (which range up to $\sim10^{-3}~\text{Am}^{2}\,\text{kg}^{-1}$) (Borlina et al. 2021; Fu et al. 2014), the bulk remanent field of a 100-km radius asteroid composed of randomly oriented 0.1-mm radius magnetized chondrules is expected to be many orders of magnitude below the sensitivity of the Magnetometer even at Orbit D (Polanskey et al. 2023).

Bulk chondrite NRM was acquired after accretion of the parent body during low-temperature crystallization (Cournède et al. 2015) and/or thermal metamorphic events (Bryson et al. 2019b; Carporzen et al. 2011; Gattacceca et al. 2016). This NRM is unidirectional across meteorites up to at least the hand sample scale (up to $>10$ cm). The source of this field has been mainly attributed to either a nebular field (if the meteorites were magnetized while the nebula was still present) (Fu et al. 2021) or an internal core dynamo in a partially differentiated body (Bryson et al. 2019b; Carporzen et al. 2011; Elkins-Tanton et al. 2011; Weiss et al. 2012; Weiss and Elkins-Tanton 2013; Maurel et al. 2021). Amplification of the IMF by conducting asteroid interiors (Anand et al. 2022; O’Brien et al. 2020) and by impact-generated plasmas (Muxworthy et al. 2017) have also been recently proposed as a source for carbonaceous chondrite NRM. However, the first mechanism has difficulty explaining the intensity of the observed NRM given the temporal variability of the IMF (Oran et al. 2018) and the second is challenged by the spatial uniformity of the peak laboratory demagnetization temperatures observed amongst meteorite subsamples (Gattacceca et al. 2016). Only the external field sources could be spatially uniform across a planetary body (e.g., Runcorn 1975) and so could conceivably produce uniform magnetization at the $\sim200$-km scale (Courville et al. 2022).

#### Iron and Stony-Iron Meteorites

Given that many iron and stony-iron meteorites typically cooled and underwent subsolidus recrystallization several Ma to hundreds of Ma after the dissipation of the solar nebula, this would leave a core dynamo as the most likely magnetizing field. However, it had long been assumed that iron and stony-iron meteorites formed in metallic cores that were largely isothermal due to thermally insulatation by thick, overlying silicate mantles. In such a scenario, by the time they cooled through the Curie temperature, the entire core would already be solid and therefore any dynamo would have ceased. Therefore, it was expected that iron meteorites would not contain ancient NRM (Cisowski 1987). However, over the last 15 years, it has been realized that many iron meteorite parent bodies either underwent catastrophic collisions that stripped away part of their silicate mantles (Matthes et al. 2020; Yang and Goldstein 2006; Yang et al. 2008, 2010), or alternatively contained reservoirs of metal within their silicate mantles located at shallower depths than their core (Bryson et al. 2015; Maurel et al. 2020; Nichols et al. 2021, 2016). On such planetesimals, meteorites could have met the conditions required for NRM acquisition while their parent body’s core dynamo was still active (Neufeld et al. 2019; Scheinberg et al. 2015).

In iron and stony-iron meteorites, reliable paleomagnetic records can be carried by microstructures called cloudy zones (Bryson et al. 2014a,b; Einsle et al. 2018; Mansbach et al. 2022; Maurel et al. 2019; Uehara et al. 2011). Cloudy zones are ensembles of $<200$-nm-diameter, ferromagnetic “islands” that acquire their NRM during their crystallographic transformation into the mineral tetrataenite at its $320~^{\circ}\text{C}$ ordering temperature. A high spatial resolution, synchrotron-based technique called X-ray photoemission electron microscopy (XPEEM) (Scholl 2002) has recently been adapted to measure cloudy zone magnetizations (Bryson et al. 2014b). XPEEM magnetic measurements rely on the preferential absorption of X-rays depending on the orientation of the sample’s local magnetization to obtain an average magnetization vector of the cloudy zone. Since 2014, XPEEM has enabled the study of a diversity of iron and stony-iron meteorite parent bodies. In particular, recent studies of IVA irons, IAB irons, IIE irons and main group (MG) pallasites collectively demonstrate that like stony meteorite parent bodies, iron and stony iron meteorite parent bodies had a diversity of differentiation states and magnetic field environments. These studies illustrate the wide range of possibilities for Psyche to have experienced and retained a record of an ancient magnetic field. We review each of these in turn.

**Fig. 6:**
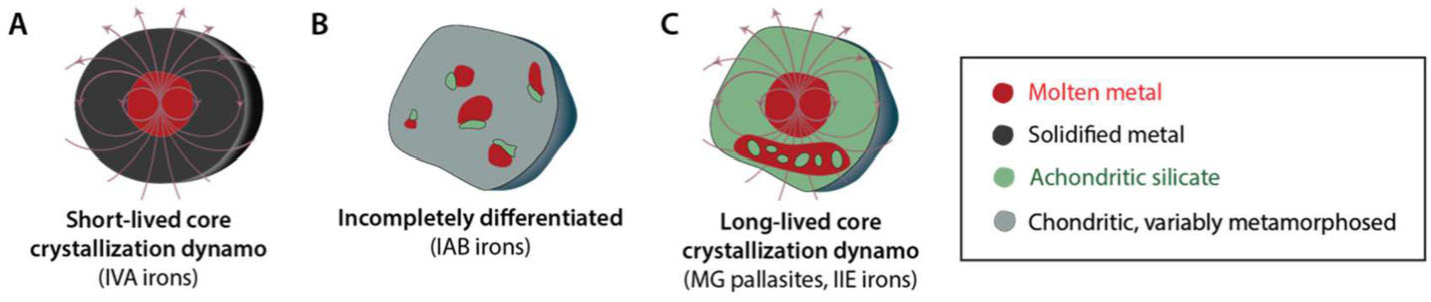
Magnetic histories inferred from paleomagnetic studies of iron and stony iron meteorites. ($\mathbf{A}$) Magnetization in main group pallasites and IIE iron meteorites suggests that their parent bodies generated a dynamo and formed a molten metallic core. Mineralogical and chemical studies separately indicate that that their parent body was differentiated. These meteorites could have cooled through their Curie points during the dynamo lifetime and record these field if they formed in metal pools trapped within the crust. ($\mathbf{B}$) Lack of magnetization in IAB iron meteorites suggests their body may not have generated a dynamo. Mineralogical and chemical studies suggest that their parent body was partially differentiated. ($\mathbf{C}$) Remanent magnetization in IVA irons is consistent with formation in the crust of an inwardly crystallizing, mantle stripped metallic planetesimal. Such a model was first motivated by the cooling rate and Ni content data. See text for references

Essentially all known IVA irons, IIE irons, and MG pallasites likely cooled too slowly to record a nebular magnetic field, so any NRM would likely be a record of dynamo. Cooling rate studies of IVA irons indicated that their parent body likely lost most of its silicate mantle through impacts occurring early in its history (Moskovitz and Walker 2011; Yang et al. 2008). Combined cooling rate and major element analyses indicate that the exposed core likely crystallized from the outside-in, akin to one of the scenarios envisioned for Psyche (left side of panel A of Fig. 1). An XPEEM study of the IVA meteorite Steinbach found that its cloudy zones carry NRM attributed to a $\gg50$-μT dynamo field powered by advection of the IVA core (Bryson et al. 2017). The fact that such a mantle-stripped core could generate a dynamo field, recorded by its outermost layers, is supported by the numerical simulations (Neufeld et al. 2019) mentioned in Sect. 1.3 (Fig. 6 panel A).

The IAB iron meteorites are thought to have orginated from at least three parent bodies (Worsham et al. 2017). Two parent bodies (sampled by the MG-sLL and sLM-sLH subgroups) are thought to either have undergone only localized differentiation (Hunt et al. 2018) or have remained undifferentiated and experienced multiple metal-pool forming impacts (Worsham et al. 2017). XPEEM analyses of one sLL and one sLH IAB iron meteorite found that neither of these meteorites carried significant NRM (Fig. 6 panel B) (Bryson et al. 2014b; Nichols et al. 2018).

Both the MG pallasites and the silicate-bearing IIE irons likely formed via impacts that mixed exogenous or endogenous metal with silicates in their parent bodies’ mantles (Bryson et al. 2015; Maurel et al. 2020, 2021; Nichols et al. 2021, 2016; Tarduno et al. 2012). These events led to the meteoritic metal cooling through the ordering temperature while core dynamos were active (Fig. 6 panel C).

Among five MG pallasites analyzed with XPEEM, two reached the ordering temperature prior to 120 Ma after accretion and do not carry significant NRM (Nichols et al. 2016). In contrast, the other three later-cooled meteorites were magnetized by a field with an intensity between 2 and 200 μT (Bryson et al. 2015; Nichols et al. 2021). The MG pallasites therefore appear to have captured the onset of a dynamo. These data are consistent with a dynamo powered by core crystallization (Nimmo 2009) whose initiation appears to have been delayed until $>100$ Ma after parent-body accretion when the core cooled to its liquidus temperature.

Three IIE iron meteorites were analyzed with XPEEM and found to have cooled in a field between 5 and 300 μT (Maurel et al. 2020, 2021). Their NRM are dated to $\sim80$, $\sim100$ and $\sim160$ Ma after CAI-formation using ^40^Ar/^39^Ar geochronology (Bogard et al. 2000). These late ages and high paleointensities essentially rule out all magnetic field sources except that of a dynamo powered by core crystallization.

### Asteroid Magnetospheres

The dynamo magnetic field intensities inferred from meteorite paleomagnetic studies are in some cases sufficiently high that such ancient dynamos would have formed magnetospheres, regions surrounding the bodies dominated by their internal magnetic field from which external plasmas like the solar wind are excluded. Here we examine whether the remanent field produced by the NRM in asteroids could lead to the formation of a present-day magnetosphere. A minimum condition for the formation of a magnetosphere is that the body’s equatorial remanent magnetic field, $B_{\mathrm{MP}}$, generates a magnetic pressure $P_{\mathrm{B}} = B_{\mathrm{MP}}^{2} /2 \mu _{0}$, that is sufficiently strong to balance the dynamic pressure of the solar wind, $P_{\mathrm{sw}} = \rho _{\mathrm{sw}} v_{\mathrm{sw}}^{2}$, at the subsolar point of the body’s surface [see Gombosi 2009]. Here, $v_{\mathrm{sw}}$ and $\rho _{\mathrm{sw}} = m_{\mathrm{H}} n_{\mathrm{sw}}$ are the solar wind bulk velocity and density, $n_{\mathrm{sw}}$ is the solar wind number density, $m_{\mathrm{H}}$ is the mass of the hydrogen atom, and $\mu _{0} $ is the permeability of free space. At the magnetopause, the equatorial field of a spherical, uniformly magnetized Psyche with magnetization, $m_{\mathrm{P}}$, is $B_{\mathrm{MP}} =F \mu _{0} m_{\mathrm{P}} R_{\mathrm{P}}^{3} /(9 r_{\mathrm{MP}}^{3} )$ where $F\sim4$ is the upstream field enhancement relative to that of a dipole in a vacuum (Gombosi 2009). Setting $r_{\mathrm{MP}} = R_{\mathrm{P}}$ in the equation $P_{\mathrm{sw}} = P_{B}$ gives

1 $$ m_{\mathrm{P}} > v_{\mathrm{sw}} \sqrt{\frac{18\rho _{\mathrm{sw}}}{\mu _{0} F}} $$

For a Psyche-sized body and using $v_{\mathrm{sw}} = 400~\text{km}\,\text{s}^{-1}$ and $n_{\mathrm{sw}} = 1~\text{cm}^{-3}$ (Gombosi 2009), this gives a minimum magnetization $m_{\mathrm{P}} \sim 0.04~\text{A}\,\text{m}^{-1}$ and a minimum moment of $M_{\mathrm{P}} \sim 2\times 10^{14}~\text{Am}^{2}$. Therefore, coincidentally, the minimum detectable moment specified by the level 1 requirement (Sect. 1.3) would just barely produce a magnetosphere and exclude solar wind protons with mean solar wind velocities (gyroradius $\sim150$ km) and would easily exclude electrons (gyroradius $\sim5$ km). By comparison, a uniformly magnetized Psyche-like body with the maximal magnetization found in iron meteorites (e.g., moment of $2\times10^{17}~\text{Am}^{2}$; Sect. 1.3) would have $r_{\mathrm{MP}} \sim 5~R_{\mathrm{P}}$.

Equation (1) provides a first order means to predict the size of the magnetosphere on its dayside. Three-dimensional modeling of the interaction is needed to appropriately plan how Magnetometer data would be used to determine the magnetization. The large planetary magnetospheres in the solar system are typically simulated using the magnetohydrodynamic equations, the system of equations combining the hydrodynamic Euler equations and Maxwell’s equations of electromagnetism and neglecting the displacement current. This approximation allows us to treat the solar wind plasma as a fluid, greatly increasing computational efficiency.

However, because the solar wind electron gyroradius but not proton gyroradius is small compared to the size of the minimum magnetosphere, only the electrons but not the protons can be treated with a fluid prescription, making the magnetohydrodynamic equations inaccurate. Rather, the protons must be modeled such that their gyration motion is resolved using particle-in-cell methods. These methods solve the equation of motion of “macroparticles” representing a large number of particles as an approximation of the true particle distribution function (Sugiyama and Kusano 2007). However, such codes are much more computationally expensive than fluid codes: in a fluid code representing a quasi-neutral electron and proton plasma, the domain is divided into cells for which only eight variables are solved (the fluid density, pressure, three components of the velocity and three components of the magnetic field). Running full kinetic codes for three-dimensional global simulations is not feasible on any existing supercomputing platform and this will likely remain the case for the coming years (Gombosi et al. 2021).

Systems whose lengthscale falls between the electron and proton gyroradii can be simulated by a hybrid computational model in which protons are modeled with a particle-in-cell approach and the electrons are treated as a fluid (Kallio et al. 2008). Because even such hybrid codes are expensive, most numerical models of asteroid magnetic fields have used either three-dimensional magnetohydrodynamic codes (Baumgartel et al. 1997; Blanco-Cano et al. 2003; Omidi et al. 2002) or 2–2.5 dimensional hybrid codes (Baumgartel et al. 1994; Blanco-Cano et al. 2003, 2004; Omidi et al. 2002; Wang and Kivelson 1996). Despite these approximations, these simulations broadly validated condition (1) (e.g., Blanco-Cano et al. 2004 and Omidi et al. 2002). More recently, three-dimensional hybrid codes have modeled asteroids with permanent moments at or above the upper limit expected for Psyche (Fatemi and Poppe 2018; Oran et al. 2022; Simon et al. 2006).

**Fig. 7:**
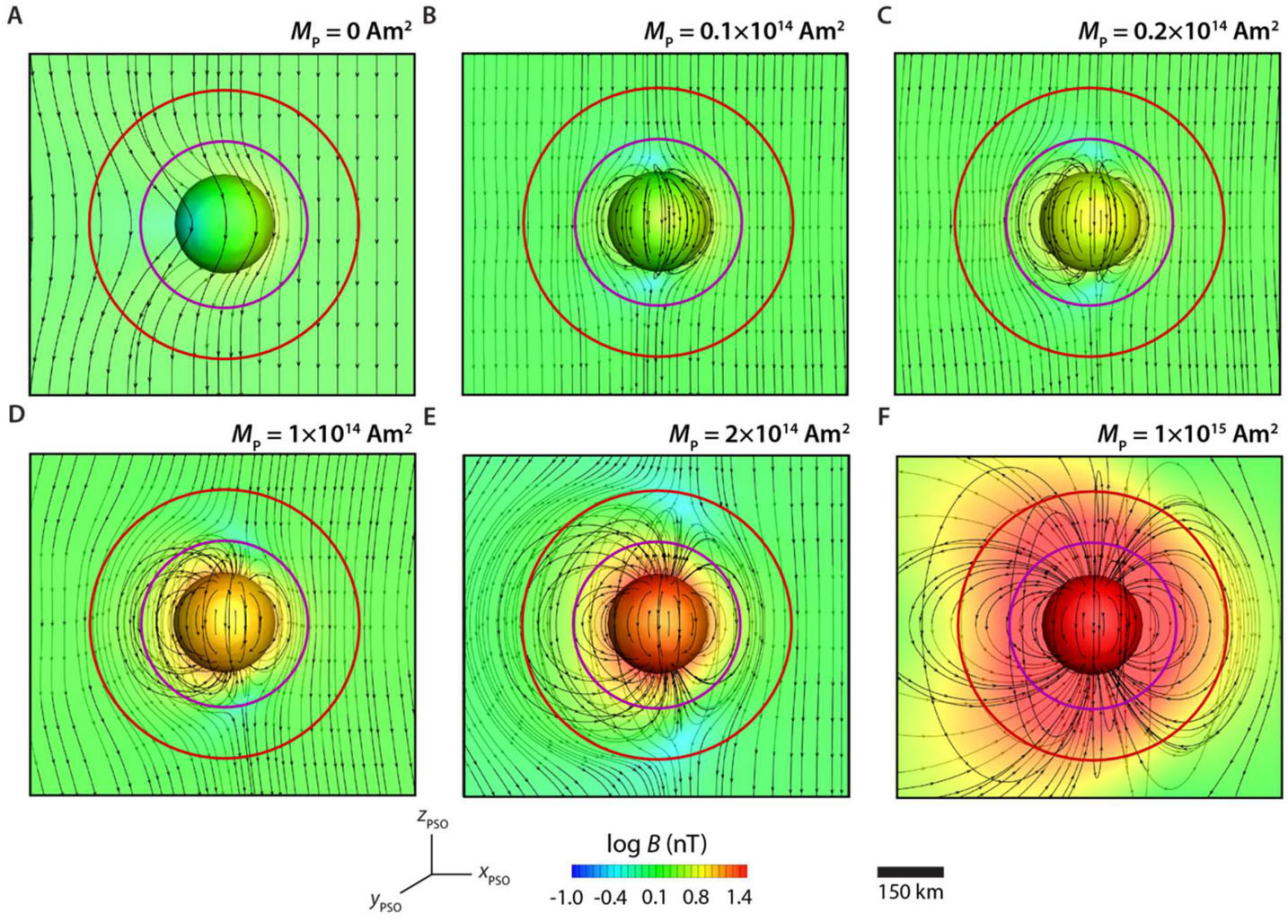
Hybrid simulations showing the magnetic field of a perfectly conducting spherical body of radius 111-km with a remanent magnetic moment of ($\mathbf{A}$) 0, ($\mathbf{B}$) $0.05\times $, ($\mathbf{C}$) $0.1\times $, ($\mathbf{D}$) $0.5\times $, ($\mathbf{E}$) $1\times $ and ($\mathbf{F}$) $5\times $ the level 1 requirement moment ($2\times10^{14}~\text{Am}^{2}$). The simulation is performed in the PSO frame. The body’s magnetic moment is aligned along the spin axis and oriented toward $+z$ direction. The solar wind is flowing toward the $-x$ direction and the IMF is aligned along the $+z$ direction. The solar wind has a density of 1 particle $\text{cm}^{-3}$, a speed of $400~\text{km}\,\text{s}^{-1}$, an IMF magnitude of 1 nT, an electron temperature of 100,000 K and a proton temperature of 40,000 K. These are typical conditions for slow wind streams in the asteroid belt (Fatemi and Poppe 2018; Oran et al. 2022). The simulations were performed using the HYB code (Kallio et al. 2008). Each panel shows a spherical surface representing the surface of the body and a plane passing through the day-night plane ($x$–$z$ plane), both colored by the magnetic field magnitude. The magnetic field topology is traced by black curves. The 190 and 75 km mean altitudes of the nominal Orbits C and D are shown as red and purple circles, respectively. The scale bar shows the size of the proton gyroradius in the nearby undisturbed IMF. Compasses show orientation of PSO coordinate system

We have conducted new three-dimensional hybrid simulations of electrically conducting Psyche-like bodies with lower remanent magnetization (following the approach described in Oran et al. 2022) (Fig. 7). These simulations show that Eq. (1), which corresponds to the moment of the simulation shown in Fig. 7E, slightly underestimates the minimum moment: a true magnetosphere that excludes protons and electrons occurs at moments $>\sim10^{15}~\text{Am}^{2}$ (Fig. 7F). We also see that for moments between $\sim10^{13}$ and $\sim10^{14}~\text{Am}^{2}$, a volume with closed magnetic field lines forms around the body, but only electrons are excluded from the region (Fig. 7B-E). Finally, for unmagnetized, conducting bodies, there is only a weak perturbation to the IMF near the body and no region of closed field lines (Fig. 7A).

### Past Asteroid and Comet Magnetic Field Measurements

Six previous missions acquired magnetic measurements in the vicinity of ten asteroids. All but two of these asteroid encounters were flybys. No clear indication of a remanent magnetic field was obtained for these bodies, such that these missions only provided upper limits on the asteroids’ magnetic moments (Table 2). A signature of an internal field can be an observed as an increase of the ambient magnetic field compared to the IMF as the spacecraft approaches the object and/or as changes in the magnetic field and turbulence levels due to the change in plasma environment as the spacecraft enters a magnetosphere (Greenstadt 1971).

**Table 2:** Summary of past missions constraining asteroidal magnetic fields

Body	$m_{\mathrm{s}}$ (Am^2^ kg^−1^)	Estimation method	Caveats	Encounter	CA (body radii/km)	References
(951) Gaspra	<10^−2^	IMF rotation + modeling	Signature could be solar wind feature (Blanco-Cano et al. 2003)	Galileo 10/29/1991 Flyby	230/1600	Baumgartel et al. (1994) and Kivelson et al. (1993)
(243) Ida	–	IMF rotation + modeling	Signature is a solar wind feature (Blanco-Cano et al. 2003)	Galileo 8/28/1993 flyby	169/2700	
(253) Mathilde	–	No detection		Near-Earth Asteroid Rendezvous (NEAR) 7/27/1997 flyby	46/1212	Cheng (2002)
(433) Eros	<1.9 × 10^−6^	No conclusive detection, upper limit only		NEAR 12/23/1998 landing	0/1	Acuña et al. (2002)
(9969) Braille	<10^−2^	Magnetometer data fit to possible dipole	Signature suspected to be spacecraft noise	Deep Space 17/28/1999 flyby	36/28	Richter et al. (2001)
(2867) Šteins	<10^−5^–10^−3^	No detection, upper limit only		Rosetta* 9/5/2008 flyby	160/800	Auster et al. (2010)
(21) Lutetia	<5.9 × 10^−7^	No conclusive detection, upper limit only		Rosetta* flyby	62/3120	Richter et al. (2012)
(4) Vesta	<10^−5^	No magnetometer, upper limit only		Dawn Orbit	0.4/210	Villarreal (2018)
(1) Ceres	–	No magnetometer, detection of energetic particles consistent with a bow shock	Bow shock may be due to a transient atmosphere	Dawn 6/27/2015 Orbit	4.7/4400	Russell et al. (2016)
(162173) Ryugu	<10^−6^	No conclusive detection, upper limit only	Hayabusa2 Mobile Asteroid Scout 6/27/2018 (arrival) 7/11/2019 (landing)	0/1	Herčik et al. (2020)	

*Notes:* The first column lists the object name. The second column lists the upper limit on the specific magnetization, $m_{\mathrm{s}}$, as deduced from the observations. The third column described the method used to constrain the magnetization in the second column. The fourth column lists any caveats to the detection method. The fifth column lists the mission, date of the encounter, and type of encounter. The sixth column lists the altitude of closest approach (CA) during the encounter (0 for missions that ended in a landing) in units of body radii and km. For elongated bodies, the equivalent radius or mean radius were used. For Ceres, this column lists the distance at which energetic particles were detected (not the mission’s CA). The last column lists the reference for the information

*For the Rosetta mission, two planned flybys of (140) Siwa and (4979) Otawara were planned (Pätzold et al. 2001) but were cancelled during the mission

At large distances where the spacecraft remains outside the magnetopause, a signature of the magnetosphere would be manifested as draping of the IMF around the obstacle (e.g., Fig. 7). In this case, one can deduce the existence of an obstacle from comparing disturbances in the IMF far from the body with theoretical predictions of the interaction of the body with the solar wind, as was done for (951) Gaspra (Baumgartel et al. 1994; Kivelson et al. 1993). This indirect method can be used to place an upper limit on the magnetic field (see Table 2) but such draping can be either due to NRM or, if the asteroid is conductive, to induced magnetic fields. If the asteroid is indeed unmagnetized but conductive, the Magnetometry Investigation may be able to constrain its internal conductivity profile (Elkins-Tanton et al. 2020) which would constrain its composition (metallic versus stony). Finally, there are indirect methods of inferring the presence of an intrinsic magnetic field that do not rely on magnetometry data such as the presence of a bow shock, as was done in the case of the Dawn mission orbiting (1) Ceres (Russell et al. 2016).

## The Psyche Magnetometer

### Introduction

**Fig. 8:**
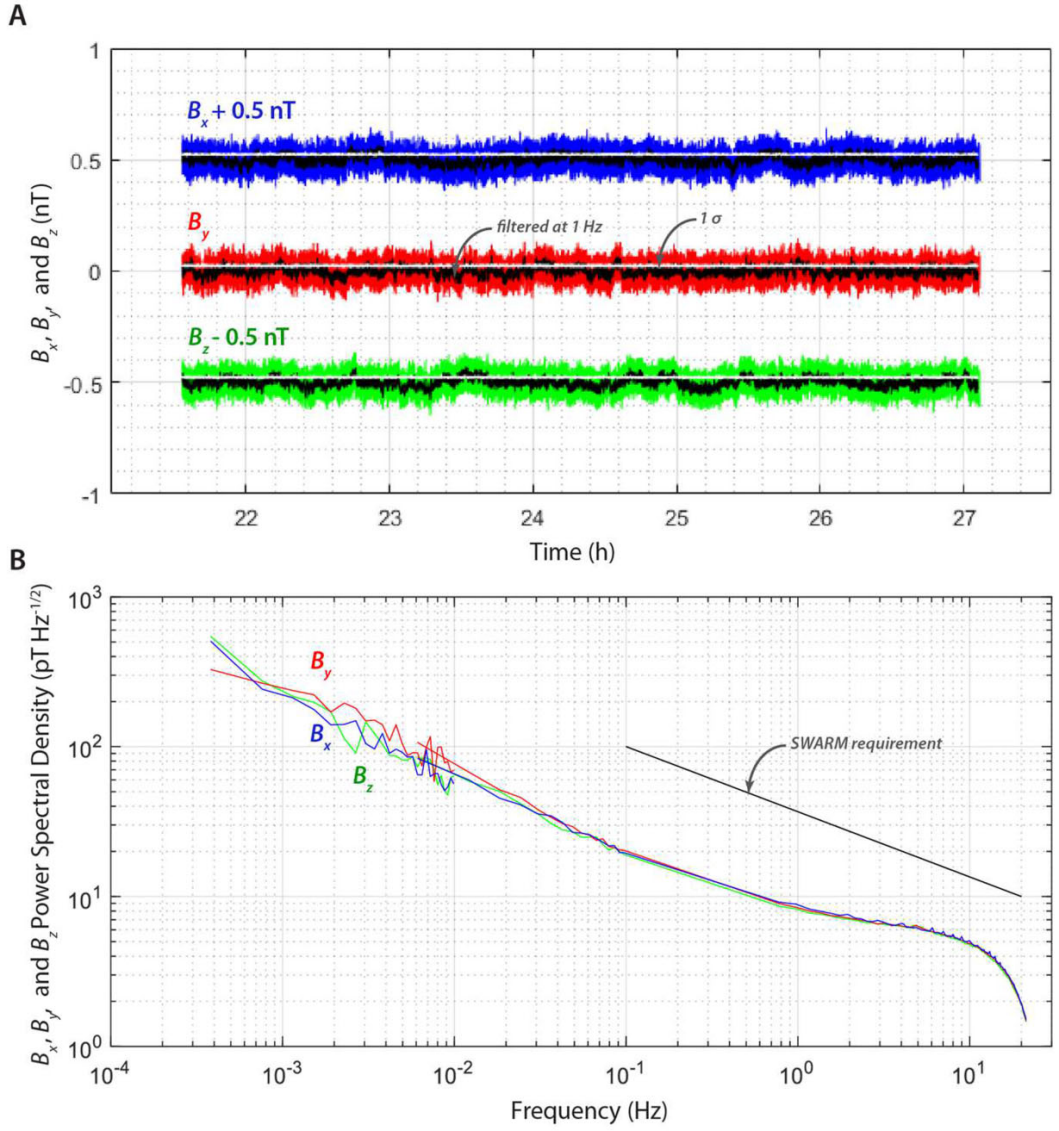
End-to-end noise of Flight Model 2 (SU2 paired with EU2 side A) ($\mathbf{A}$) Time series of three field components $B_{x}$ (blue), $B_{y}$ (red), and $B_{z}$ (green). $B_{x}$ is offset by 0.5 nT and $B_{z}$ is offset by −0.5 nT for clarity. ($\mathbf{B}$) Power spectral density (PSD) of $B_{x}$ (blue), $B_{y}$ (red), and $B_{z}$ (green). Shown for comparison was the noise requirement for the Swarm Mission Vector Field Magnetometer, which provides heritage for the Psyche Magnetometer

The Magnetometer was designed and built by DTU. It has high heritage from the Vector Field Magnetometer (VFM) onboard the European Space Agency’s Swarm mission (2013–) (Merayo et al. 2008) with the main changes being an expanded range, additional qualification to cope with cryogenic temperatures expected during flight and shock and random vibrations during launch, as well as additional electronics radiation protection. Each field component is measured with a single dynamic range of $\pm80{,}000$ nT with a noise of $<30~\text{pT}\,\text{(Hz)}^{-1/2}\,\text{axis}^{-1}\,3\sigma $ at 1 Hz ($<39~\text{pT}\,\text{axis}^{-1}\,3\sigma $ integrated over 0.1–1 Hz) (Figs. 3 and 8). The VFM in turn has extensive heritage and in particular is the main instrument in missions mapping the magnetic field of the Earth: Ørsted (1999–) (Nielsen et al. 1995) and the Challenging Microsatellite Project (CHAMP) (2000–2010) (Merayo et al. 2005).

DTU designed, built, and delivered engineering models and ultimately three Flight Models for the Psyche project. Flight Models 1 and 2 became the pair SU1 and EU1 and the pair SU2 and EU2, respectively. Flight Model 3 is held in reserve at JPL for anomaly resolution and as a backup flight spare.

### Fluxgate Magnetometry

The Magnetometer uses the fluxgate principle for measuring magnetic fields (Primdahl 1979). The basis of this technique is that a high permeability material (ring core) is periodically driven in and out of saturation by a toroidally-wound excitation coil wrapped with pickup coils. If an ambient field is present, the part of the toroid wrapped with a pickup coil oriented parallel to the ambient field will remain saturated for a longer duration than the part of the toroid wrapped with a pickup coil oriented antiparallel to the ambient field. The flux induced in the pickup coils detects this temporal asymmetry in the saturation state which can subsequently be detected as a second harmonic in the pick-up coils. Finally, compensation coils act to maintain zero field in the pickup coils, serving as a null detector such that when the external field changes, the compensating current is accordingly adjusted to bring the sensor into balance.

The Psyche Magnetometer’s core consists of 11 wraps of an amorphous magnetic metal ribbon, which is by heat treatment optimized for several magnetic properties (e.g., nearly zero magnetostriction and magnetic hysteresis) (Nielsen et al. 1997, 1990). As result of this treatment, the sensor has superior performance in applications where thermal and long-term stability are driving parameters. The excitation coil with a current at a frequency of 15.625 kHz drives the core into saturation twice per cycle. The pickup coil is short-circuited by an operation amplifier (Primdahl et al. 1991) producing the fluxgate signal at 31.25 kHz (second harmonic of the excitation frequency), which together with all even harmonics are subsequently demodulated and fed to an integrator. The output of the integrator is connected through a feedback resistor to the compensation coil, which defines the magnetic axis. The voltage across the feedback resistor is digitized by an effective 23.5-bit analog-to-digital converter with a resolution of $\sim11$ pT.

### Gradiometry

As discussed above, SU1 and SU2 are mounted along a boom pointing along the FS $+z$-axis at distances of 1.45 m and 2.15 m from the $+Z$ spacecraft deck (Fig. 4). This enables the differenced field between these two positions to be computed from the measured field at each sensor, $\vec{B}_{\mathrm{M}} ( \vec{r} )= ( B_{x}, B_{y},B_{z} )$:

2 $$ \Delta \vec{B} = \vec{B}_{\mathrm{M}} ( \vec{r}_{2} ) - \vec{B}_{\mathrm{M}} ( \vec{r}_{1} ) $$

where $\vec{r}$ is the position relative to the spacecraft and subscripts 1 and 2 refer to field measurements at SU$n $ for sensor number $n = 1{:}2$.

The measured field, $\vec{B}_{\mathrm{M}} = \vec{B}_{\mathrm{amb}} + \vec{B}_{\mathrm{FS}} + \vec{B}_{\mathrm{N}} $, for the ambient field of scientific interest, $\vec{B}_{\mathrm{amb}}$, and the instrument noise, $\vec{B}_{\mathrm{N}}$. Furthermore, the flight system field, $\vec{B}_{\mathrm{FS}} = \vec{B}_{\mathrm{FS},\mathrm{DC}} + \vec{B}_{\mathrm{FS},\mathrm{AC}}$ for flight system steady and time-variable fields $\vec{B}_{\mathrm{FS},\mathrm{DC}}$ and $\vec{B}_{\mathrm{FS},\mathrm{AC}}$. Now, the ambient field (e.g., the asteroid’s remanent field) is expected to be constant at the two SUs because $r_{2} - r_{1}$ is small relative to their distances from the ambient field source (e.g., the asteroid). By comparison, the field of the nearby flight system will be stronger at SU2 compared to SU1. Therefore, the differenced field is only a function of $\vec{B}_{\mathrm{FS}}$ and $\vec{B}_{\mathrm{N}}$:

$$\begin{aligned} \Delta \vec{B} &= \vec{B}_{\mathrm{FS}} ( \vec{r}_{2} ) + \vec{B}_{\mathrm{amb}} ( \vec{r}_{2} ) + \vec{B}_{\mathrm{N},2} - \bigl[ \vec{B}_{\mathrm{FS}} ( \vec{r}_{1} ) + \vec{B}_{\mathrm{amb}} ( \vec{r}_{1} ) + \vec{B}_{\mathrm{N},1} \bigr] \\ &= \vec{B}_{\mathrm{FS}} ( \vec{r}_{2} ) - \vec{B}_{\mathrm{FS}} ( \vec{r}_{1} ) + \vec{B}_{\mathrm{N},2} + \vec{B}_{\mathrm{N},1} \end{aligned}$$

where $\vec{B}_{\mathrm{N},2}$ and $\vec{B}_{\mathrm{N},1}$ are the instrument noise for measurements with SU1 and SU2. For a given flight system component, the ratio of the $a$^th^ component of the flight system field at SU1 and the $b$^th^ component of the flight system field at SU2 is known as the coupling matrix $\alpha _{ab} = B_{\mathrm{FS}, a} ( r_{1} ) / B_{\mathrm{FS}, b} ( r_{2} )$. If is $\boldsymbol{\alpha} $ known a priori, then an estimate of the ambient field can be obtained from (Ness et al. 1971):

3 $$ \vec{B}_{\mathrm{amb}}' = ( 1- \boldsymbol{\alpha} )^{-1} \bigl[ \vec{B}_{\mathrm{M}} ( \vec{r}_{1} ) - \boldsymbol{\alpha} \vec{B}_{\mathrm{M}} ( \vec{r}_{2} ) \bigr] $$

If $\boldsymbol{\alpha} $ is not known a priori, it can be estimated by fitting the differenced field with a dipole estimate, $\vec{M}_{\mathrm{FS}}'$ (or in principle, multipole) representing the flight system field:

4 $$ \vec{B}_{\mathrm{FS}} ( \vec{r} ) = \frac{\mu _{0}}{4\pi} \biggl[ \frac{3 \boldsymbol{r} ( \vec{M}_{\mathrm{FS}}' \boldsymbol{\cdot} \vec{r} )}{r^{5}} - \frac{\vec{M}_{\mathrm{FS}}'}{r^{3}} \biggr] $$

where here the origin is assumed to be located at the dipole location.

The spacing of the Psyche SUs along the boom was determined to optimize the use of gradiometry to characterize and remove the spacecraft fields from the measured field. Our detailed approach is described in Cochrane et al. (2023) [see their approach for two sensors]. We conducted simulations to estimate the uncertainty in knowledge of the flight system field as a function of spacing between the sensors. Using a multiple dipole model of the flight system field (Sect. 3.6.3), we modelled the flight system DC and AC field at each sensor as a function of their spacing along the boom. SU1 was fixed at the end of the boom while SU2 was sequentially moved along the length of the boom. We then used the predicted fields at the two SUs to estimate the moment and position of a dipole (six total parameters) representing the flight system, $\vec{M}_{\mathrm{FS}}'$. We repeated this for 1,000 instances of the flight system field configuration in which the orientations of the dipoles in the multiple dipole flight system model were randomly varied. We found that when SU2 is too close to the spacecraft deck, the offset dipole approximation [Eq. (4)] is a poor approximation of the flight system field. At the other extreme, when SU2 is too close to SU1, the difference between their two measurements becomes comparable or less than the sensor noise [i.e., $\vec{B}_{\mathrm{FS}} ( \vec{r}_{2} ) - \vec{B}_{\mathrm{FS}} ( \vec{r}_{1} ) << + \vec{B}_{\mathrm{N},2} + \vec{B}_{\mathrm{N},1}$]. This resulted in the identification of a range of optimum positions for DC field removal between 1.35 m and 1.65 m from the spacecraft deck and for AC and step removal between 1.15 and 1.5 m from the spacecraft deck. A location near the outer edge of the optimum positions for AC removal was selected. Considerations for this choice included placing SU2 as far from the spacecraft as possible to allow for graceful degradation in case SU1 failed while still optimizing spacecraft field removal with the gradiometer.

### Description of Instrument

**Fig. 9:**
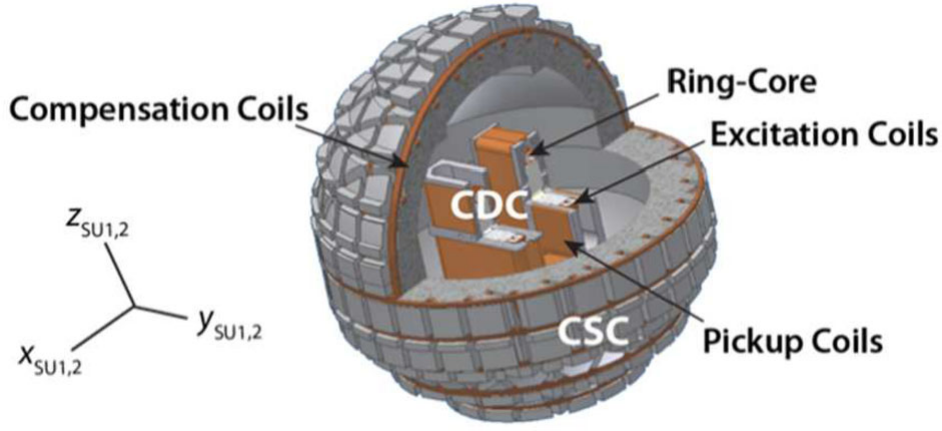
Cutaway of a Sensor Unit (SU). Shown is the CSC (grey sphere wrapped with compensation coils) and interior CDC (wrapped ring core wrapped with pickup coils). Compass shows orientation of SU1 and SU2 coordinate systems

Three ring core elements in an orthogonal configuration and wrapped with excitation and pickup coils form the Compact Detector Coils (CDC). In principle, the pickup coils could also used for compensating the external field, such that the CDC could be used as a fluxgate magnetometer with excellent performance. However, the nonspherical shape of its pickup coils limits the omnidirectional linearity (Brauer et al. 1997) (Fig. 9). In particular, a non-linearity in the range of Earth’s field is present due to the proximity of the detector coils along each of the three axes, which mutually interfere due to stray fields produced during compensation. This problem is overcome with the addition of the surrounding Compact Spherical Coils (CSC), whose spherical shapes create an internal field that is nearly zero (Primdahl and Jensen 1982) (Fig. 9). The excitation of the three excitation coils are series coupled and therefore share the excitation drive, whereas the detector and compensation coils have each their own circuitry. The sensor elements are carefully adjusted as to reduce as much as possible the feedthrough (Petersen et al. 1992) for optimal signal to noise ratio. As a result the uncertainty associated with interactions between the axes (omni-nonlinearity knowledge) for each SU is only $24~\text{ppm}\,3\sigma $, which translates to $0.24~\text{pT}\,\text{axis}^{-1}\,3\sigma $ for a measured field of 10 nT (Fig. 3).

The Magnetometer is equipped with a field-programmable gate array (FPGA) that controls all digital functions in the instrument: it produces a highly stable excitation square wave signal with a frequency of 15.625 kHz and a detection signal of frequency 31.25 kHz that are used by the excitation drive and phase detector blocks, respectively. After power on, the instrument operates autonomously. It activates the phase detector with respect to the excitation current in the sensor using the pre-flight predetermined default parameters for the frequency, delay and width, and transmits data at up to 50 Hz.

### Pre-flight Calibration and Expected Performance

The Magnetometer design incorporates components with high thermal and temporal stability against degassing in space, stresses from changing temperatures, and total ionizing dose from high energy particles. Nevertheless, its performance is ultimately limited by tolerances in the machining processes, electronic parts and assembly of the individual parts and their temperature dependencies. A calibration process has been implemented to correct for these effects. The raw analog-to-digital converter readings from the Magnetometer are transmitted through spacecraft telemetry to ground, where they are converted from engineering units (eu) to magnetic field values (nT) as discussed in Sect. 4. This requires knowledge of the offset vector, $\vec{O}_{n}$, the scale factor matrices, $\boldsymbol{S}_{\boldsymbol{n}}$, the orthogonality matrices, $\boldsymbol{V}_{\boldsymbol{n}}$, and the rotation matrices, $\boldsymbol{R}_{\boldsymbol{n}}$, that transform the orientation of the data from the individual SU1 and SU2 frames to the FSe and other frames. Note that in addition to depending on the SU-EU number, $n$, $\vec{O}_{n}$ and $\boldsymbol{S}_{\boldsymbol{n}}$ also depend on the choice of EU side (A or B); for simplicity, we have omitted a subscript denoting the latter dependency here and below.

Because conversion from eu to nT occurs during ground data processing, the Psyche Magnetometry uncertainty budget does not depend on the values of the coefficients in $\vec{O}_{n}$, $\boldsymbol{R}_{\boldsymbol{n}}$, $\boldsymbol{S}_{\boldsymbol{n}}$ and $\boldsymbol{V}_{\boldsymbol{n}}$ but rather on our knowledge of them. Therefore, we conducted a pre-flight calibration campaign at DTU to measure these parameters based on the methods described in Merayo et al. (2000a,b). We also have designed an in-flight validation campaign that will yield estimates of the sum of $\vec{O}_{n}$ and the flight system DC field at each sensor throughout the mission (Sect. 5).

The parameters $\vec{O}_{n}$, $\boldsymbol{S}_{\boldsymbol{n}}$ and $\boldsymbol{V}_{\boldsymbol{n}}$ are slightly temperature and time-dependent. For this reason, each SU contains two temperature sensors (located in the CDC and CSC) and each EU incorporates a third temperature sensor. These have an accuracy of less than $0.27~^{\circ}\text{C}\,3\sigma $ in the operating temperature range following ground calibration. The Magnetometer transmits these temperatures, which are then used in the ground data processing.

Offset knowledge as a function of temperature is the dominant term in our Instrument Uncertainty and is specified by a driving requirement for the Magnetometer design (Fig. 3). Both the SU and EU contribute to the offset. These were characterized independently with pre-flight tests in which the SU and EU were scanned separately in temperature. The SU offset drift was measured such that after calibration, our knowledge of its contribution to the offset is better than 0.25 nT over the whole operational temperature range of the SUs ($\sim-125$ to $+50~^{\circ}\text{C}$) (e.g., Table 3). For the much smaller temperature ranges that the SUs are expected to experience during Orbit D ($\pm1~^{\circ}\text{C}$), the repeatability of the offset is better than $0.15~\text{nT}\,\text{axis}^{-1}\,3\sigma $ after calibration (Fig. 3). The knowledge of the EUs’ contribution after calibration is better than 0.2 nT for their whole operational temperature range (−20 to $+50~^{\circ}\text{C}$) (e.g., Table 3) and just 0.01 nT over the temperature range they will experience in Orbit D.

**Table 3:** Magnetometer mass, volume and power

Magnetometer component	Final mass (g)	Final size (mm)	Power (W)
SU1 including harness and bracket	896	CSC diameter: 82	
		bracket diameter 140	
SU2 including harness and bracket	892	CSC diameter: 82	
		bracket diameter 140	
EU1	924	100 × 124 × 66	2.2
EU2	923	100 × 124 × 66	2.2

*Note:* The first column lists the Magnetometer component, the second column lists its mass, the third column lists its dimensions, and the fourth column lists its power. Power is for operation in near-zero field and $0~^{\circ}\text{C}$ like that expected for level 1 requirement Psyche dipole moment in orbit. Operation in an 80 μT field at $70~^{\circ}\text{C}$ would increase the power to 2.6 and 2.3 W for EU1 and EU2, respectively

Based on heritage from the Swarm VFM, the long-term temporal drift of the Magnetometer offset is expected to be exceptionally low (well below $58~\text{pT}\,\text{axis}^{-1}\,\text{y}^{-1}\,3\sigma $). Given our regular in-flight zero-level calibration activities (Sect. 5), it should contribute negligibly to the Instrument Uncertainty (Fig. 3). In particular, for Psyche with a moment equal to that of the level 1 requirement (Sect. 1.3), the spacecraft would pass within the asteroid’s magnetosphere at altitudes below $\sim170$ km (just below that of the nominal orbit C) where it would be shielded from the solar wind. We currently estimate that the spacecraft will spend 402 days at or below this altitude, which sets the maximum time since the last estimate of the Magnetometer zero-level from monitoring of solar wind fluctuations (Sects. 4.4 and 5.3).

Calibration of the scale factor as a function of temperature was also conducted for both the SUs and EUs. The scale factor drift of the sensor depends uniquely on changes in the volume of the CSC shell, which has an isotropic thermal expansion rate of $30~\text{ppm}\,^{\circ}\text{C}^{-1}$. The scale factor of the EUs is on the order of $1~\text{ppm}\,^{\circ}\text{C}^{-1}$. After compensation, the uncertainty on the measurement due to the residual effect is better than $3.1~\text{pT}\,\text{axis}^{-1}\,3\sigma $ in a 10 nT field (Fig. 3).

Due to the design of the Magnetometer, the nonorthogonality angles are constant with temperature. After ground calibration, knowledge uncertainty on these angles is better than $15~\text{arcsec}\,3\sigma $ (i.e., $0.73~\text{pT}\,\text{axis}^{-1}\,3\sigma $ for a 10 nT field) (Fig. 3), consistent with extensive data from heritage instruments (Yin and Lühr 2011). The main contribution to field orientation uncertainty is knowledge of the SU axes relative to the FS frame which totals $0.17^{\circ}\,3\sigma $ (i.e., $30~\text{pT}\,\text{axis}^{-1}\,3\sigma $ for a 10 nT field).

### Accommodation

#### Resources

The total masses of each of the two EUs and each of the two SUs including their brackets and harnesses are 0.92 g and 0.9 g, respectively. The length of the intra-harness between each EU and SU is selectable without degrading the performance and for Psyche was required to be 3.85 m for all Flight Models. The masses of each harness and bracket are $120~\text{g}\,\text{m}^{-1}$ and 135 g, respectively. Finally, the mass of each CSC, which has a diameter of 82 mm, is 310 g. The size of each EU is $100\times 124\times 66$ mm. The power dissipation of each Flight Model is about 2.2 W (due to a higher radiation hardened DC-DC converter). Table 3 provides details.

#### Interfaces

To mount the SU onto the spacecraft, a carbon fiber reinforced polymer bracket was developed with a finger shape, with one end glued onto the CSC so as not to deform the sensor’s structure during temperature changes. The other end of the bracket has a plate with four holes used to attach the sensor to a Ti node on the boom. The EU has a standard interface with four bolts into the panel of the spacecraft. Attitude transfer from the SU axes to the FS coordinate system was achieved by a reference cube assembly temporarily mounted on each SU. The orientations of the three normal faces of the cube were determined with respect to the SU coil axes following the triaxial coil method of Primdahl et al. (2002). During assembly onto the spacecraft, the rotation matrices relating the two reference cubes and the spacecraft were measured. After this, the reference cube assemblies were dismounted.

Upon application of power to one of the EU sides (A or B), that side will connect to the SU autonomously. Each SU was designed to be nonredundant as it does not contain any electronic components. Each EU side contains all resources to operate independently and it is not expected that both sides will be powered on at the same time during science operations. The EU chassis walls are composed of aluminum with side wall thicknesses and top and bottom wall thicknesses of 3 mm and 6 mm, respectively. This provides radiation protection to the EUs at a level that is about 10 times larger than the expected total ionizing dose (Oran et al. 2022).

Although the Magnetometer can operate with a wide range of power supply voltages, for Psyche the nominal voltage is 31 V (with a low and high interval of 27.2 and 33.6 V, respectively). Table 3 shows the power consumption of the units. To avoid current loops that could create disturbing magnetic fields, the power connector provides the nominal voltage and return, but the grounding of the unit is performed through a stud that is connected separately to the reference grounding point of the spacecraft power supply. The grounding of the SU harness is also carefully separated and routed as to reduce any interference between the two SU-EU pairs or any other equipment near the location of the Magnetometer hardware.

Using a low-voltage differential signaling (LVDS) electrical layer, the Magnetometer communication features a universal asynchronous receiver-transmitter (UART) serial interface at 115,200 bps with a structure of 1 start bit followed by 8 bits and then a stop bit. Data and commands are organized in packets following the Swarm Packet Utilization Standard (PUS) protocol (European Cooperation for Space Standardization 2003). A few seconds after power on, the Magnetometer enters the default mode (50 Hz sampling) and begins to transmit science data at a rate of approximately 1 s where each packet contains 50 vectors in addition to time information, temperature and other flags that provide the status of the instrument. To synchronize the science data, a pulse-per-second (PPS) signal is provided to the instrument, which is used to relate the time of the first sample of each packet with respect to this signal with an interval of 125 μs. The end-to-end time characterization is verified on the ground and is typically 0.2 ms. A spacecraft time message (STM) is sent to the Magnetometer every second enabling the Magnetometer to report the information needed to fully recover the absolute time of the data on the ground. Should the PPS or STM be absent, the Magnetometer will propagate the time from the last time it was synchronized to them since being powered on. Housekeeping data are transmitted once per minute. Uncompressed science data packets contain 500 bytes, but if the field magnitude does not change significantly, the packet can be compressed to about 200 bytes, which yields a data volume of less than 2 kbps assuming a realistic 90% compression rate. Telecommands can be sent to the Magnetometer, but it is not expected that this will be performed in-flight apart from the power on sequence. The telecommands can also change the sampling frequency to 10 Hz, 1 Hz, and 0 Hz (the latter would just provide housekeeping data and no science data). They can also change the excitation frequency and the delay of the detector, but these are not expected to be used in-flight.

The allowable temperatures for Magnetometer components are given in Table 4. The instrument has been qualified, tested, and calibrated to this range. Importantly, the spherical shape of the CSC, which undergoes isotropic thermal expansion and contraction, means that Magnetometer does not require active thermal control in-flight despite the very wide range of temperatures experienced.

**Table 4:** Allowable flight temperatures for the SUs, EUs, and harnesses

Magnetometer component	Operational minimum (^∘^C)	Operational maximum (^∘^C)	Non-operational minimum (^∘^C)	Non-operational maximum (^∘^C)
SUs	−125	50	−125	50
EUs	−20	50	−30	50
Harnesses	−125	50	−125	50

*Note:* The first column lists the Magnetometer component, the second and third columns list the minimum and maximum operational temperatures and the fourth and fifth columns list the minimum and maximum non-operational temperatures

#### Magnetic Cleanliness

As discussed in Sect. 1.3, the dominant contribution to the Magnetometer’s Reconstructed Measurement uncertainty is the flight system’s magnetic field (Fig. 3A). Even though the science data are acquired at 50 Hz, the Investigation is sensitive to fields ranging from DC up to 100 kHz, with frequencies $>50$ Hz aliased into the measured signal. We use two methods to minimize this noise source. First, as discussed in this section, a magnetic cleanliness program was implemented to design and build a flight system that produces minimal DC and AC fields at the SUs. Second, as discussed in Sect. 5, the flight system field measured at the sensors is suppressed during ground data processing using gradiometry and solar wind monitoring.

The Psyche magnetic cleanliness program uses three broad approaches: prevention, characterization, and mitigation of flight system fields. For more details, we refer the reader to de Soria Santacruz-Pich et al. (2020). Our foremost approach was prevention: to design the flight system to minimize stray DC and AC fields. This led us to two sets of requirements. First, we require that our knowledge of the flight system field should be better than 0.4 nT per axis $3\sigma $ after suppression using gradiometry and other techniques while using both SU1/EU1 and SU2/EU2 (Sect. 4.4) (Fig. 3A). This flows down to requirements on knowledge uncertainty for fields in four frequency ranges: $<10^{-5}$ Hz, $10^{-5}$ to 0.1 Hz, 0.1 to 25 Hz, 25 Hz to 100 kHz (Fig. 3B). Second, the flight system field system from 0.1 Hz to 100 kHz should be sufficiently low that our level 1 requirement could be met even if SU1 failed and we were only left with measurements from the inboard sensor, SU2.

These metrics then drove the development of magnetic cleanliness requirements and guidelines that were then flowed to designers of spacecraft subsystems and components early in the development process. These included maximizing the distance between magnetic field sources [e.g., Hall thrusters and cold gas thrusters, components of the Deep Space Optical Communication (DSOC) payload, latch vales, and traveling-wave tube amplifiers (TWTAs)] and the SUs, minimizing the usage of ferromagnetic and high magnetic permeability materials (e.g., by using aluminum for structural supports), reducing current loops (e.g., in the solar arrays), and self-compensation of the fields within a component to minimize magnetic poles (e.g., by using back-to-back mounting of latch vales and arranging ferromagnetic struts in the DSOC in a closure configuration). We also ensured that the spacecraft has no conductive paths along which there could be strong thermal gradients that could generate thermoelectric currents during flight (e.g., Brauer et al. 2018). Because many of these mitigations are cost-neutral or low-cost, they led to major efficiencies in cost and schedule by minimizing the need for later mitigation activities.

**Fig. 10:**
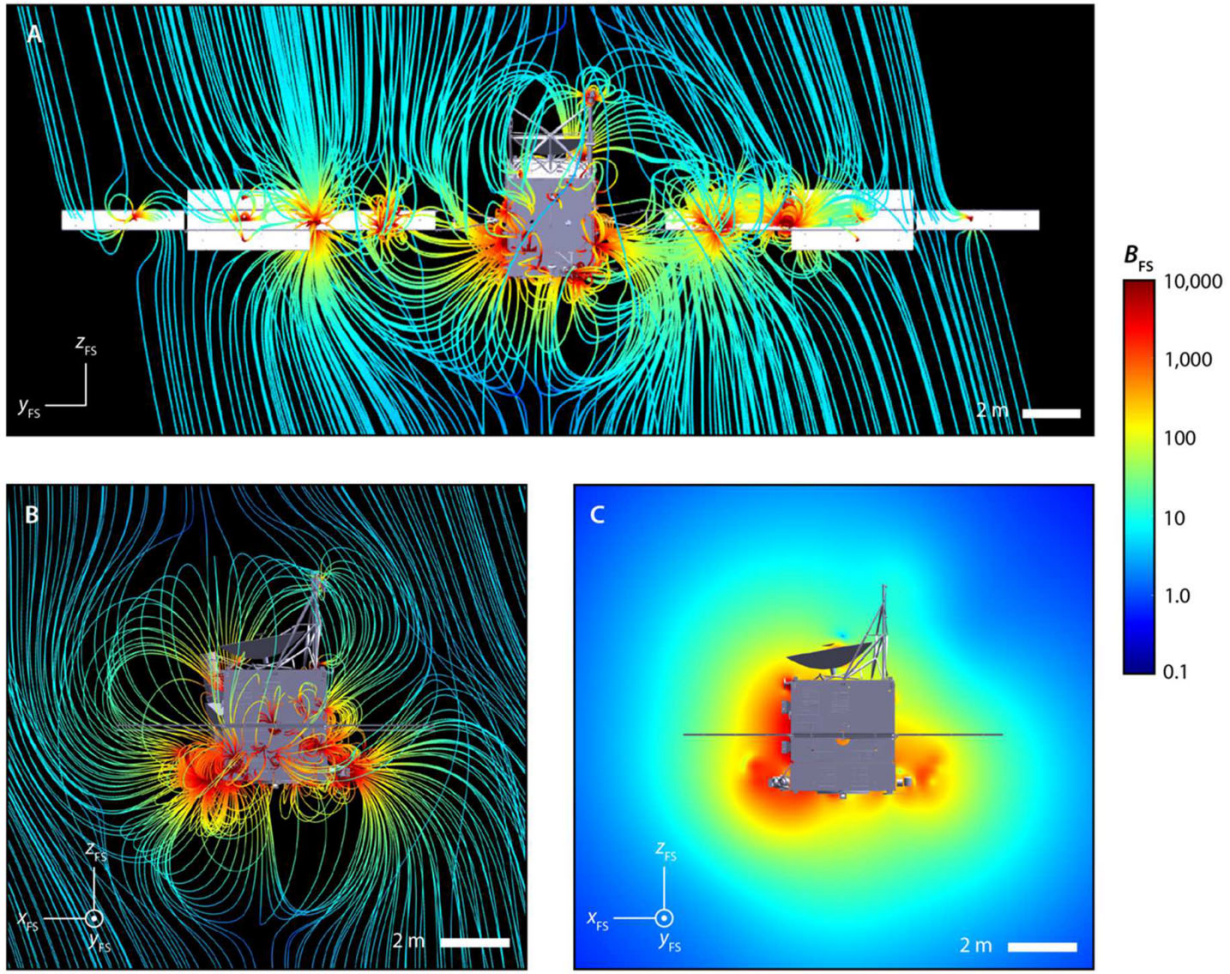
Psyche flight system magnetic field. (**A**, **B**) Two views of the modeled flight system field plus a background IMF, $\vec{B}_{\mathrm{IMF}} = (1~\text{nT}, 1~\text{nT}, 5~\text{nT})$ and superposed on the spacecraft model (grey and white). Shown are magnetic field lines simulated from more than 200 individual dipole sources using a Runge–Kutta method. The magnetic field lines are color-coded according to field strength, with blue representing weaker field strengths and red representing stronger field strengths. Note that the solar array currents are modeled by multiple dipole field point sources (**C**) Magnetic field intensity in the flight system in plane with surface normal in the $y$ direction containing the boom and two SUs. Same color intensity as in (**A**, **B**). Compasses in (**A**–**C**) show orientation of FS coordinate system

Our second approach was to characterize the AC and DC flight system magnetic field. This was conducted at the subsystem level prior to assembly of the spacecraft and at the flight system-level during assembly in the JPL high-bay. With respect to pre-assembly characterization, all spacecraft subsystems were evaluated for their impact to the magnetics budget and those considered to likely produce the strongest fields at SU2 were tested and characterized early in the development phase of the mission. In particular, the magnitudes of the magnetic moments of the prioritized subsystems were measured. Results from subsystem-level tests were used to construct a spacecraft-level magnetics model that approximates the DC and AC field using dipoles positioned at the location of each subsystem (following Mehlem 1978a,b; Cochrane et al. 2023; Mehlem and Wiegand 2010; Neubauer and Schatten 1974; Zhang et al. 2007). Measured subsystems were assigned a dipole magnitude equal to the value measured with dipole orientation in a random direction, while unmeasured subsystems were assigned dipole magnitudes estimated to be upper limits from heritage measurements of analog subsystems. This multiple-dipole model was then used to estimate the flight system field at the locations of SU1 and SU2 (Fig. 10).

The flight system-level testing involved measuring the field at multiple locations around the spacecraft and then fitting for the spacecraft net dipole moment. System-level testing was conducted as part of the campaign to characterize the electromagnetic interference and electromagnetic compatibility of the spacecraft during assembly in the high-bay at the JPL. The results (Table 5) indicate that the spacecraft is remarkably magnetically clean and will enable the Magnetometry Investigation to meet the two performance metrics discussed above.

**Table 5:** Psyche flight system magnetic field measured at JPL

Field quantity	Measured value
DC field at SU2^a^	18 nT
Ratio of DC field at two SUs^b^	1.7
AC field at SU2 integrated from 10^−5^ to $0.1~\text{Hz}$^c^	$<1.4~\text{nT}\,\text{axis}^{-1}\,3\sigma $
Ratio of AC field at SU2 integrated from 10^−5^ to 0.1 Hz at SUs^d^	>1.4
AC field at SU2 integrated from 0.1 to 25 Hz^e^	$<1.2~\text{nT}\,\text{axis}^{-1}\,3\sigma $
Ratio of AC field integrated from 0.1 to 25 Hz at SUs^f^	>2.5
AC field at SU2 integrated from 25 Hz to 100 kHz as sensed by Magnetometer^g^	$<0.4~\text{nT}\,\text{axis}^{-1}\,3\sigma $

*Note:* The first column lists the spacecraft field quantity measured and the second column lists the value measured

^a^Total DC (<10^−5^ Hz) field at the location of the inboard sensor, $B_{\mathrm{FS},\mathrm{DC}} ( r_{2} )$

^b^Ratio of the DC field at the inboard to that at the outboard sensor, $B_{\mathrm{FS},\mathrm{DC}} ( r_{2} ) / B_{\mathrm{FS},\mathrm{AC}} ( r_{1} )$

^c^Estimated maximum 3*σ* per axis variation of frequency-integrated AC field for all spacecraft subsystems at inboard sensor, $3 [ \sum_{P} \int _{10^{-5}~\text{Hz}}^{0.1~\text{Hz}} \mathrm{PSD}_{p, s} ( r_{2}, f ) \mathrm{d} f ]^{1/2}$. Here $\mathrm{PSD}_{p, s}$ is the power spectral density (nT^2^ Hz^−1^) for spacecraft subsystem, *p*, and field axis *s* = *x*, *y* or *z* and *f* is frequency

^d^Ratio of the integrated AC field from 10^−5^ to 0.1 Hz at the inboard to that at the outboard sensor, $[ \sum_{P} \int _{10^{-5}~\text{Hz}}^{0.1~\text{H}} \mathrm{PSD}_{p, s} ( r_{2}, f ) \mathrm{d} f ]^{1/2} / [ \sum_{P} \int _{10^{-5}~\text{Hz}}^{0.1~\text{Hz}} \mathrm{PSD}_{p, s} ( r_{1}, f ) \mathrm{d} f ]^{1/2}$

^e^Estimated maximum 3*σ* per axis variation of frequency-integrated AC field for all spacecraft subsystems at inboard sensor, $3 [ \sum_{P} \int _{0.1~\text{Hz}}^{25~\text{Hz}} \mathrm{PSD}_{p, s} ( r_{2}, f ) \mathrm{d} f ]^{1/2}$

^f^Ratio of the integrated AC field from 0.1–25 Hz at the inboard to that at the outboard sensor, $[ \sum_{P} \int _{0.1~\text{Hz}}^{25~\text{Hz}} \mathrm{PSD}_{p, s} ( r_{2}, f ) \mathrm{d} f ]^{1/2} / [ \sum_{P} \int _{0.1~\text{Hz}}^{25~\text{Hz}} \mathrm{PSD}_{p, s} ( r_{1}, f ) \mathrm{d} f ]^{1/2}$

^g^Estimated maximum 3*σ* per axis variation of frequency-integrated AC field for all spacecraft subsystems at inboard sensor as measured by the Magnetometer, $3 [ \sum_{P} \int _{25~\text{Hz}}^{100~\text{kHZ}} \mathrm{PSD}_{p, s} ( r_{2}, f ) R ( f )^{2} \mathrm{d} f ]^{1/2}$. Here, *R*(*f*)≤1 is the Magnetometer response function

### Operations

The nominal operations plan for the Magnetometer is simple: it will be turned on soon after launch and remain on for the remainder of the mission. All data will be sent to the ground at 50 Hz and will be processed at MIT (Sect. 4). All measurements are expected to be usable for science except those acquired during periods of thrusting by the electric propulsion system, which will occur periodically during cruise and transfer orbits, and during DSOC operations, which will occur prior to arrival at Psyche (Polanskey et al. 2023). Real-time commands (e.g., power cycling and changing fluxgate tuning parameters) are only expected to be issued to resolve anomalies. MIT will conduct science planning and JPL will develop formal commands following communications with MIT. Anomalies will be resolved through cooperation between MIT, DTU and the JPL. MIT will conduct long-term monitoring of magnetic field data, temperature measurements, and internal instrument voltages to assess instrument performance.

## Data Pipeline

### Overview of Data Levels

**Fig. 11:**
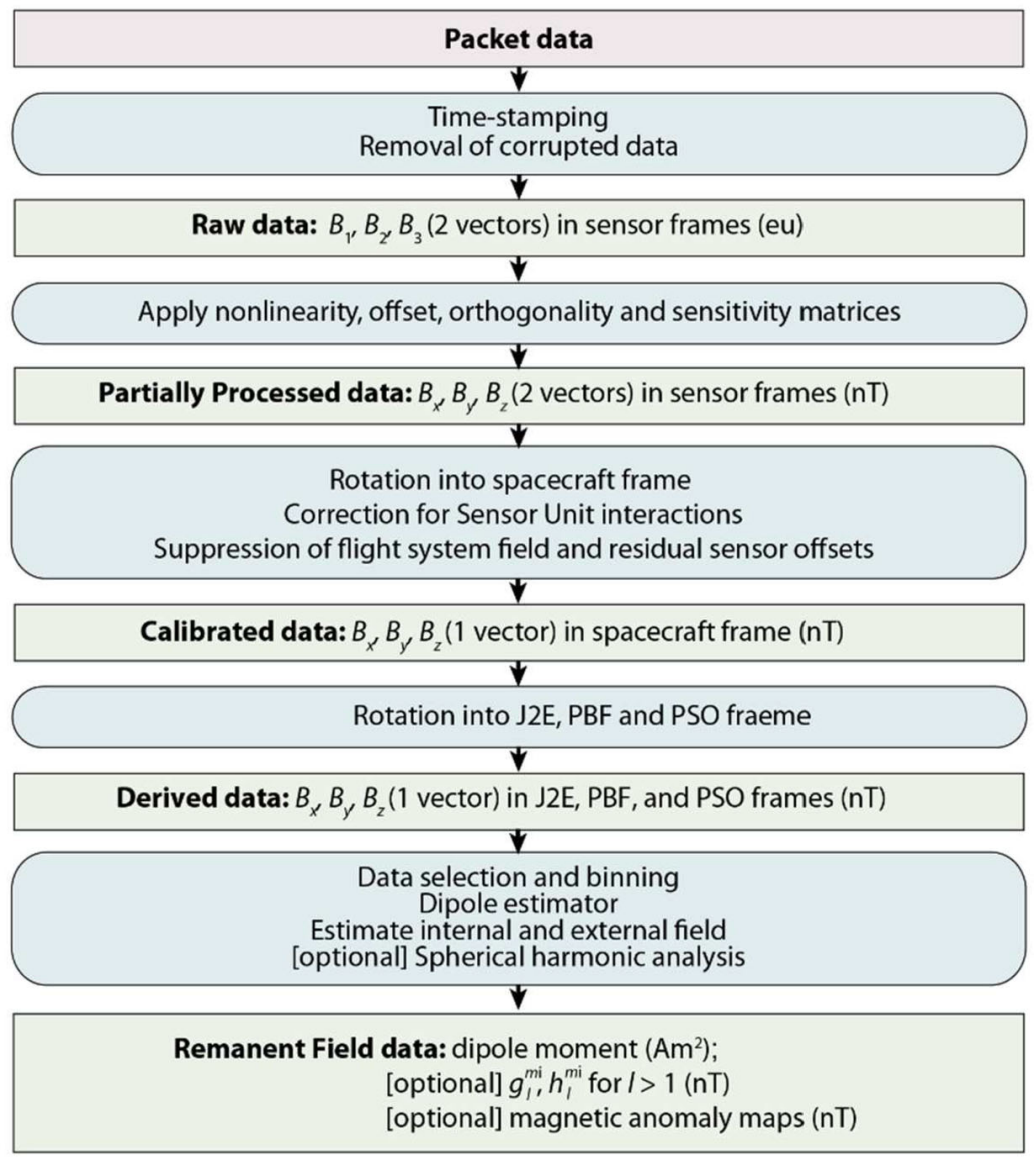
Psyche Magnetometry Investigation data levels. Packet data received from the spacecraft (pink box) are time-stamped, cleaned of corrupted data, and organized into files of approximately 1 day in duration (second blue oval) to produce Raw data: a magnetic field vector for each SU in SU coordinates with units of eu [Eq. (5)] (top green box). These are then calibrated (second blue oval) to produce Partially Processed data: a magnetic field vector for each SU in SU coordinates with units of nT [Eq. (7)] (second green box). These data are then rotated into the FS frame, flight system fields are suppressed, and residual senser offsets removed (third blue oval) to produce Calibrated data (third green box): a single magnetic field vector in the spacecraft frame with units of nT. These data are then rotated into the J2E, PBF, and PSO frames (fourth blue oval) to produce Derived data in units of nT [Eq. (9)] (fourth green box). The data are then binned and down-selected and the internal field and externally generated field are estimated (blue box). These are used to produce Remanent Field data, which at a minimum are a constraint on the asteroid’s dipole moment [e.g., Eq. (10)] (bottom green box). Optionally, if a remanent field is detected and mapped with sufficient coverage in PBF coordinates, a magnetic anomaly map and possibly also internal Gauss coefficients with degree $l > 1$ can be estimated [Eq. (12)]

The data products produced by the Psyche Magnetometry Investigation are organized into six data levels (Fig. 11): packet, Raw, Partially Processed (ppr), Calibrated (cal), Derived (der) and Remanent Field (rf) (Fig. 11, Table 6). The processing for each is presented in the following sections.

**Table 6:** Magnetometer data levels

Data level	Reference frames	Units	Description
Packet	SU1 and SU2 axes 1, 2, and 3	DN	Telemetry packets received from the instrument
Raw	SU1 and SU2 axes 1, 2, and 3	eu	Unpacked telemetry including time-tagged magnetic field and temperature measurements
Partially Processed	SU1, SU2 axes *x*, *y*, and *z*	nT, ^∘^C	Magnetic field vectors corrected for temperature dependent scale factors, offsets, non-linearity, and non-orthogonality using ground calibration data,
Calibrated	FS	nT	Magnetic field vectors with flight system field and residual instrument offets suppressed
Derived	PBF, PSO, J2E	nT	Calibrated, rotated magnetic field vectors
Remanent Field	PBF	Am^2^, nT	Estimate of dipole moment and possibly also higher-order internal Gauss coefficients and magnetic anomaly maps

*Note:* The first column lists the PDS4 data level, the second column lists the reference frame (SU1/SU2 = Sensor Unit 1/2 frames, FS = flight system, PBF = Psyche-body-fixed, PSO = Psyche-Sun-orbit, and J2E = Psyche-centered EMO J2000), the third column lists the units (DN = digital numbers, eu = engineering units), and the fourth column gives a description of the data level

Packet data are the raw telemetry sent from the instrument to the spacecraft and subsequently packaged into Consultative Committee for Space Data Systems (CCSDS) File Delivery Protocol (CFDP) data products for transmission. Science packets include three magnetic field axes and temperature measurements required for calibration and the information to accurately timestamp the measurements. The observations are nominally reported with a frequency of $f_{s} = 50$ Hz using a lossless compression scheme in which the change in the field is reported rather than the total field. These data are decompressed and time-tagged to generate the Raw data. Corrupted data are also flagged and removed at this data level.

Partially Processed data have been corrected for temperature-dependent scale factors, offsets, non-linearity and non-orthogonality using ground calibration data. The measurements are in physical units (nT and $^{\circ}\text{C}$) and are given in both the individual sensor frames (SU1 and SU2) and in the FS reference frame. These corrections are based on calibration tables developed during the ground testing campaign prior to launch (Sect. 3.5). At the packet, Raw, and Partially Processed data levels, the two SU/EU pairs are treated separately so there are two files per day (one for each SU) for these data levels.

Calibrated data have been processed to suppress contributions from flight system generated-fields and residual instrument offsets and field interactions between the two SUs. Data at this level are given in nT in the FS frame. We plan to use two main methods to suppress flight system fields and residual instrument offsets. First, measurements from both SUs will enable gradiometry methods in which the flight system field can be characterized based on its property of being stronger in intensity at the inboard relative to the outboard sensor (Ream et al. 2021). Second, monitoring variations of the solar wind (Leinweber et al. 2008) during flight will be used to correct for residual instrument offsets and the flight system DC field (see Sect. 4.4 and 5.3).

The final two data levels are used for assessing the Magnetometer level 1 requirement (see Sect. 1.3). The Derived data products consist of files which contain the Calibrated data rotated into Psyche-body-fixed (PBF), Psyche-solar-orbit (PSO), and Psyche-centered EMO J2000 (J2E) reference frames. The Remanent Field data contain at minimum an estimate of the asteroid’s dipole moment. If a remanent field is discovered and mapped with sufficient accuracy and spatial coverage, the Remanent Field data may also include higher-degree internal Gauss coefficients and possibly even a map of the remanent field (e.g., magnetic anomaly map).

We now discuss in detail the data processing flow for each of these data levels. In this discussion, we assume a nominal configuration in which SU1 has been paired with EU1 side A and SU2 with EU2 side A. Therefore, the dependency of calibration constants on EU side has not been noted explicitly below.

### Raw

The Raw processing step converts the Magnetometer data products received from the spacecraft into files of approximately 1 day in duration. The data products consist of a collection of packets transmitted each second containing 50 three-component field measurements collected by the instruments every 0.02 s. The data packets are extracted from the product files, converted to a human readable format and assembled into files containing a time interval of 1 day. The spacecraft clock (SCLK) along with internal timing for the instruments are used to time-tag the measurements.

Any packets found to be corrupted and/or are otherwise unusable will be flagged either by onboard flight software or by the MIT ground data system and will be rejected from further processing. The ground data pipeline will track the identity and timings of rejected packets. The data pipeline will further scan the data stream to find, report, and resolve, any temporal discontinuities in the data.

The absolute time of the start of each packet, $t_{\mathrm{ABS}}$, is the instrument time updated based on receipt of the last PPS, measured in SCLK ticks after Epoch. The time for each measurement within the packet relative to the start of the packet, $t_{\mathrm{MAG}}$, is given by

$$ t_{m} (c)= t_{\mathrm{ABS}} + t_{\mathrm{MAG}} (c) $$

Here

$$ t_{\mathrm{MAG}} (c)= \frac{\Delta _{\mathrm{PPS}}}{8\times 10^{6}} + t_{d} + {c} / {f_{s}} $$

where $t_{d}$ is the instrument latency as found by the timing characterization test, $\Delta _{\mathrm{PPS}}$ is the relative time of the sample with respect to the last PPS received by the Magnetometer, and $c=0{:}49$ is the index of each of the 50 measurements in the packet.

Raw data are uncalibrated and in units of eu for each of the three magnetometer axes $a = 1{:}3$ in each sensor frame, SU1 and SU2:

5 $$ \vec{B}_{\mathrm{raw},n} = \left [ \textstyle\begin{array}{c} {eu}_{{n,1}}\\ {eu}_{{n,2}}\\ {eu}_{{n,3}} \end{array}\displaystyle \right ], $$

where ${eu}_{{n,a}} = e_{{n,a}}/2^{{6.5}}$ where $e_{{n,a}}$ are the raw uncompressed measurement data.

### Partially Processed Data

The production of Partially Processed data involves converting the three axes from each SU into field vector components. This involves three steps: housekeeping calibration, linearization, and linear calibration. During the housekeeping calibration, the temperature readings for the CSC, CDC and EU are linearized and converted to $^{\circ}\text{C}$ using a 7^th^ order polynomial:

$$\begin{aligned} T_{n,i} &= C_{n,0,i} + C_{n,1,i} VET_{n,i}^{1} + C_{n,2,i} VET_{n,i}^{2} + C_{n,3,i} VET_{n,i}^{3} + C_{n,4,i} VET_{n,i}^{4} + C_{n,5,i} VET_{n,i}^{5} \\ &\quad{}+ C_{n,6,i} VET_{n,i}^{6} + C_{n,7,i} VET_{n,i}^{7} \end{aligned}$$

where $i$ denotes either the CSC, CDC, or EU temperature sensor, $C_{n,0,i}$, $C_{n,1,i}$, $C_{n,2,i}$, $C_{n,3,i}$, $C_{n,4,i}$, $C_{n,5,i}$, $C_{n,6,i}$, and $C_{n,7,i}$ are the coefficients for each temperature sensor determined by thermal tests carried out prior to integrating the instrument on the spacecraft (Sect. 3.5), and $VET_{n,i}$ is the voltage measured by each temperature sensor.

The next step is to apply a linearization algorithm to the Raw measurements:

6 $$ \vec{E}_{n} = \left [ \textstyle\begin{array}{c} E_{n,1}\\ E_{n,2}\\ E_{n,3} \end{array}\displaystyle \right ],\quad \mbox{where } E_{n,a} = \bigl[ 1+ ( k_{n,2,a} + k_{n,3,a} eu_{n,a} ) eu_{n,a} \bigr] eu_{n,a} $$

and where the linearity parameters $k_{n,2,a}$ and $k_{n,3,a}$ were determined by testing measurements against an in-house high-performance voltmeter.

Finally, linear calibration applies the orthogonality matrix, temperature dependent offset vector, and sensitivity matrix to determine the applied magnetic field vector in nT in the orthogonalized sensor measurement frame coordinates ($x,y,z$). The measurements are calibrated using

7 $$ \vec{B}_{\mathrm{ppr}, n} = \boldsymbol{V}_{\boldsymbol{n}} \boldsymbol{S}_{\boldsymbol{n}} ( \vec{E}_{n} - \vec{O}_{n} ) $$

The offset (units of eu) is:

$$\begin{aligned} &\vec{O}_{n} = \left [ \textstyle\begin{array}{c} O_{n,1}\\ O_{n,2}\\ O_{n,3} \end{array}\displaystyle \right ],\\ &\quad \mbox{where } O_{n,a} = O_{n,0,a} + O_{n, \mathrm{CDC},a} T_{n, \mathrm{CDC}} + \Delta O_{n, \mathrm{CDC},a} ( T_{n, \mathrm{CDC}} ) + O_{n,\mathrm{EU},a} T_{n, \mathrm{EU}} + O_{n,t,a} t_{L} \end{aligned}$$

and where $T_{n, \mathrm{CDC}}$ is the CDC temperature for SU1 or SU2, $T_{n,\mathrm{EU}}$ is the temperature of EU1 or EU2, $t_{L}$ is the time in years since the launch of the spacecraft, $O_{n,0,a}$, $O_{n, \mathrm{CDC},a}$, $O_{n, \mathrm{EU},a}$ and $O_{n,t,a}$ are calibration coefficients determined prior to launch (Sect. 3.5) and $\Delta O_{n, \mathrm{CDC},a} ( T_{n, \mathrm{CDC}} )$ is a look-up table for correcting minor deviations from the temperature-dependent calibration also determined prior to launch (Sect. 3.5). The scale factor matrix $\boldsymbol{S}_{\boldsymbol{n}}$ (units of $\text{nT}\,\text{eu}^{-1}$) is:

$$ \boldsymbol{S}_{\boldsymbol{n}} = \left [ \textstyle\begin{array}{c@{\quad}c@{\quad}c} S_{n,1} & 0 & 0\\ 0 & S_{n,2} & 0\\ 0 & 0 & S_{n,3} \end{array}\displaystyle \right ],\quad \mbox{where } S_{n,a} = S_{n,0,a} + S_{n, \mathrm{CSC},a} T_{n, \mathrm{CSC}} + S_{n, \mathrm{EU},a} T_{n, \mathrm{EU}} + S_{n,t,a} t_{L} $$

for calibration coefficients $S_{n,0,a}$, $S_{n, \mathrm{CSC},a}$, $S_{n,\mathrm{EU},a}$, and $S_{n,t,a}$ determined prior to launch (Sect. 3.5). The orthogonalization matrix

$$ \boldsymbol{V}_{\boldsymbol{n}} = \left [ \textstyle\begin{array}{c@{\quad}c@{\quad}c} 1 & - \sin u_{n,1} & - \sin u_{n,2}\\ 0 & \cos u_{n,1} & - \sin u_{n,3}\\ 0 & 0 & \sqrt{1- \sin ^{2} u_{n,2} - \sin ^{2} u_{n,3}} \end{array}\displaystyle \right ] $$

where the angles, $u_{n,1}$, $u_{n,2}$, and $u_{n,3} $ are determined on the ground during instrument testing (Sect. 3.5). While the offsets and sensitivity matrix values are dependent on SU temperatures, EU temperatures and time, the non-orthogonality is constant over the operational temperature range and the lifetime of the mission.

### Calibrated Data

The Calibrated data processing begins by rotating the Partially Processed data from each SU frame into the common FS frame:

8 $$ \vec{B}_{\mathrm{cal},\mathrm{FS}, n} = \boldsymbol{R}_{n} \vec{B}_{\mathrm{cal}, n} $$

for rotation matrices, $\boldsymbol{R}_{n}$.

The rotated data can then be processed to suppress flight system fields. Several such methods are under development. We consider pure tones in the three lowest frequency bins described in Sect. 3.6.3. Additionally, we separately consider broad-spectrum flight system signals associated with step changes in the quasi-static field to a new DC value. These four categories are defined based on the methods available to identify and remove their field signatures (Table 7). As described next, the suppression of signals in each category is associated with a separate processing step. These processing steps can be conducted in any order; the following description arbitrarily follows the order starting with DC fields, to $10^{-5}$ to 0.1 Hz fields, to to >0.1 Hz fields, and finally to step changes.

**Table 7:** Flight system field frequency ranges and expected mitigation approaches

Frequency range, *f* (Hz)	Mitigation technique
<10^−5^	solar wind monitoring (when not in magnetosphere) and time-domain gradiometry (when in magnetosphere)
10^−5^ to 0.1	solar array look-up tables
>0.1	gradiometry using frequency-domain filtering
Step changes	time-domain gradiometry

*Note:* The first column lists the frequency range of pure tones (first three rows) and step changes in the DC field (fourth row) and the second column lists the associated mitigation technique for suppressing the flight system field

Estimated flight system DC fields and instrument offsets remaining after the production of Partially Processed data, $\vec{B}_{\mathrm{zl}, n}$, will be suppressed to removed the zero-level corrected field:

$$ \vec{B}_{\mathrm{cal},\mathrm{zl},n} = \vec{B}_{\mathrm{cal},\mathrm{FS}, n} - \vec{B}_{\mathrm{zl}, n} $$

We estimate $\vec{B}_{\mathrm{zl}, n}$ in two different ways. When the spacecraft is immersed in the solar wind, we will use estimates of the flight system DC field at each SU derived from monitoring Alfvénic fluctuations of the IMF (termed solar wind monitoring). These DC field estimates will be periodically derived from regular calibration activities during cruise (Sect. 5.3). Second, if and when the spacecraft enters a magnetosphere around (16) Psyche, the spacecraft would then be shielded from the solar wind (e.g., Fig. 7E, F) and solar wind monitoring would then no longer be feasible. In this case, we will estimate the flight system DC field using time-domain gradiometry. The last available zero-level field level calculations for each instrument will be used as an estimate for the spacecraft field at the locations of the SUs. These values can either be removed directly from the measurements, or, as discussed in Sect. 3.3, a coupling matrix $\boldsymbol{\alpha} $ associated with this estimate can then be constructed and the ambient field estimate using Eq. (3).

The dominant noise source in the $10^{-5}$ to 0.1 Hz range is expected to be from slow rotation of the solar panels, which generate magnetic fields mainly due to DC photoelectric currents. Therefore, as described in Sect. 5.4, another calibration activity will be to generate tables estimating contributions to $\vec{B}_{\mathrm{FS}}$ at each SU from the solar panels as a function of their orientation, $\vec{B}_{\mathrm{SA}, n} ( \theta _{S} )$, where $\theta _{S}$ is the angle of the solar array normal with respect to the spacecraft bus. These will be based on data collected during spacecraft rolls. The solar array-generated field values at each SU will be subtracted from measurements at each sensor:

$$ \vec{B}_{\mathrm{cal},\mathrm{zl},\mathrm{SA},n} = \vec{B}_{\mathrm{cal},\mathrm{zl},n} - \vec{B}_{\mathrm{SA}, n} ( \theta _{S} ). $$

Estimated flight system >0.1 Hz fields will be identified and removed using frequency-domain filtering of gradiometry measurements (Ream et al. 2021). In particular, intervals containing flight system >0.1 Hz fields can be identified when a simultaneous change occurs in the rolling maximum and minimum (upper and lower envelope) of the differenced field, $\Delta \vec{B}$. For each such interval containing >0.1 Hz field noise, a power spectrum of the differenced field is calculated. This enables identification of flight system-generated spectral peaks by selecting frequencies with power in the differenced field Fast Fourier Transform (FFT) above a specified threshold (e.g., 98^th^ percentile of amplitude values). These contributions can then be suppressed by reducing the power in the identified frequencies to the noise level (Ream et al. 2021). Because the differenced field does not contain ambient field fluctuations, this filtering will not affect the spectral peaks of the time-variable ambient fields. An inverse FFT is then used to recreate a cleaned time series for each sensor. This yields:

$$ \vec{B}_{\mathrm{cal},\mathrm{zl},\mathrm{SA},\mathrm{FDF},n} = f_{\mathrm{FDF}} ( \vec{B}_{\mathrm{cal},\mathrm{zl},\mathrm{SA},n} ) $$

Step changes in the flight system fields are expected when components that generate dominantly DC fields (e.g., heaters) turn on and off and when subsystems switch between different modes (e.g., the Multispectral Imager, high gain antenna, and power distribution assembly). These changes will be identified in two ways. First, pattern-recognition algorithms can identify rapid field changes in time series of the differenced field like those expected for near-instantaneous changes in the power state of the flight system. The algorithm currently implemented in the data pipeline uses an 0.1 s running average of the differenced field $\Delta\vec{B}$ and checks for changes in the field greater than twice the instrument noise. Coupling coefficients can be derived from simultaneously observed field changes at each sensor. Second, time-tagged spacecraft ancillary data can be used to independently identify step changes from known spacecraft subsystems. In this case, coupling coefficients can be derived during initial checkout calibration activities (Sect. 5.2). When a step change is identified it is removed from the applicable interval for each instrument:

$$ \vec{B}_{\mathrm{cal},\mathrm{zl},\mathrm{SA},\mathrm{FDF},\mathrm{SC}, n} = f_{\mathrm{SC}} (\vec{B}_{\mathrm{cal},\mathrm{zl},\mathrm{SA},\mathrm{FDF},n} ) $$

The step removal and peak suppression algorithms can be iterated to further clean the data. After these calibration steps are complete—rotation into FS coordinates and suppression of flight system fields and residual instrument offsets—and the final differenced field is determined to be less than then the acceptable noise threshold, the final result is calibrated, cleaned, science-grade data, $\vec{B}_{\mathrm{cal}}$.

### Derived Data

**Fig. 12:**
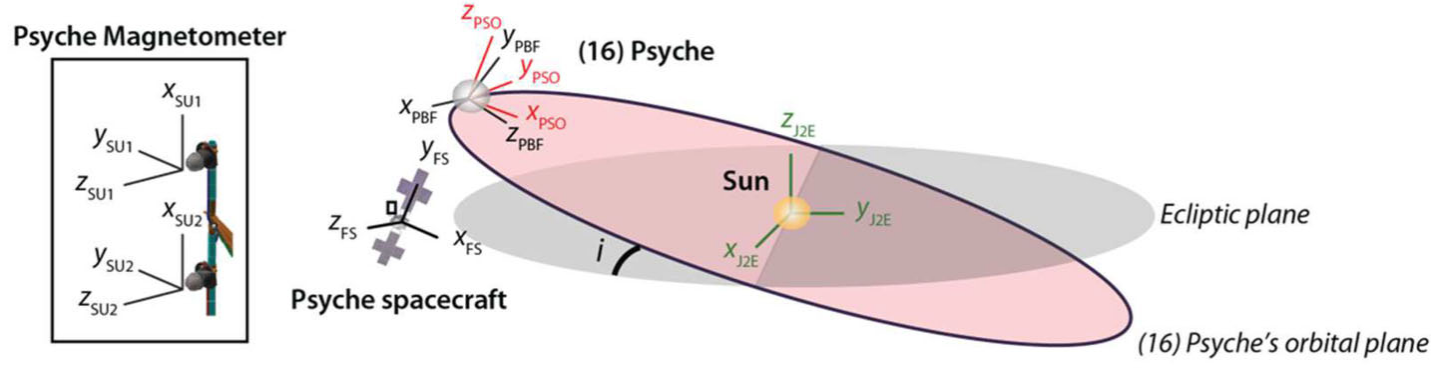
Coordinate frames used for creating Derived data products. The pink ellipse signifies the orientation of (16) Psyche’s orbital plane with respect to the ecliptic plane (gray shaded ellipse). The Sun (yellow sphere) is the center of the J2E frame (green arrows). The PSO and PBF frames (red and black arrows, respectively) are centered on the body (gray sphere) and are translated and rotated with respect to the inertial frame together as the body moves in its orbit. The PSO and PBF frames rotate with respect to each other due to both the body’s spin and the motion of the body with respect to the Sun. The Psyche flight system (Fig. 4) defines the FS frame and the individual SUs define the SU1 and SU2 frames (Figs. 4, 5, 9)

The Derived data are magnetic field measurements rotated from the FS frame into coordinate frames oriented relative to (16) Psyche’s orbit, a frame rotating with (16) Psyche, and the Sun (Fig. 12):

9 $$ \vec{B}_{\mathrm{der}, q} = \boldsymbol{R}_{q} \vec{B}_{\mathrm{cal}} $$

where $q = \text{``J2E''}$, “PBF”, or “PSO”. We discuss each of these frames below.

The J2E frame is the same as the J2000 Earth mean orbit inertial frame but centered at (16) Psyche’s instantaneous location:

- The $x$-axis is the intersection of Earth’s equatorial plane and the ecliptic plane as it was oriented at the epoch J2000, at which time it pointed to the first point of Aries.
- The $z$-axis points in the direction perpendicular to Earth’s orbital plane.
- The $y$-axis completes a right-handed system.

The PBF frame is a non-inertial frame centered at the center of mass of Psyche and rotating with the body:

- The $z$-axis is aligned with Psyche’s rotation axis.
- The $x$-axis connects the origin with the body’s prime meridian (currently defined in Shepard et al. 2021), crossing the surface at the body’s equator.
- The $y$-axis completes a right-handed system.

The PSO frame is an inertial frame centered at the center of mass of Psyche

- The $x$-axis points to Sun.
- The $z$-axis is perpendicular to the orbital plane of the asteroid.
- The $y$-axis completes a right-handed system.

The spacecraft ephemerides, as well as the ephemerides of solar system bodies are provided by the Navigation and Ancillary Information Facility (NAIF) at JPL in the J2E, PBF, and PSO frames. The data pipeline uses the SPICE toolkit to extract the information in both frames for the spacecraft trajectory and performs conversions of the magnetic field from the spacecraft frame (NAIF name “PSYC”) to the other frames as needed for different products.

The PBF frame will be used for mapping the magnetic field with respect to geographical location on the body’s surface, while the PSO frame will be used to map the field with respect to the magnetosphere, which rotates both with the body and with the direction of the incoming solar wind flow. All conversions between the frames are time-dependent and rely on knowledge of the ephemerides of Earth, (16) Psyche, the Sun, and the spacecraft, as well as the spacecraft attitude, as provide by NAIF.

### Remanent Field Data

#### Overview of Remanent Field Data

The purpose of high-level data processing is to determine whether Psyche contains remanent magnetization consistent with formation by a dynamo paleofield, and if so, to constrain the strength and pattern of this magnetization. This will yield at least one and up to three different data products. First, we will constrain the asteroid’s dipole moment and, in particular, establish whether it exceeds the threshold specified by the level 1 requirement that would indicate Psyche has magnetization like that known for iron meteorites at spatial scales of 40 km or greater. If a remanent dipole moment is detected, then depending on the ratio of the remanent field to the noise and on how spatially complete are the measurements, we will optionally create a magnetic anomaly map and possibly even estimate higher-order multipole field terms.

#### Dipole Moment

Given that the flight system magnetic field is suppressed during the production of $\vec{B}_{\mathrm{cal}}$ data, the $\vec{B}_{\mathrm{der}}$ data are expected to be dominated by contributions with sources external to the spacecraft (from hereon we drop the $q$ subscript that denotes the frame of the data). This flight system-cleaned field can be as the sum of a field internal to a radial distance $r_{P} = r_{0}$ from (16) Psyche’s center-of-mass that encloses the entire asteroid, $\vec{B}_{\mathrm{der}, \mathrm{i}}$, and a field generated external to this distance, $\vec{B}_{\mathrm{der},\mathrm{e}} $:

$$ \vec{B}_{\mathrm{der}} ( r_{0}, \theta ,\phi )= \vec{B}_{\mathrm{der}, \mathrm{i}} + \vec{B}_{\mathrm{der}, \mathrm{e}} $$

for Psyche-centric colatitude, $\theta _{P}$, and longitude, $\phi _{P}$. The main contributions to $\vec{B}_{\mathrm{der},\mathrm{i}} $ are the asteroid’s remanent field and fields due to currents induced inside the body by the changing IMF. The main contributions to $\vec{B}_{\mathrm{der},\mathrm{e}} $ are the IMF and fields due to current systems created by the interaction of the magnetized body with the solar wind.

The dipole moment of the asteroid will be estimated from $\vec{B}_{\mathrm{der}}$ data. Our hybrid simulations (Fig. 7) indicate that if $M_{\mathrm{P}}\gtrsim 1.5\times10^{14}~\text{Am}^{2}$ or $\lesssim2\times10^{13}~\text{Am}^{2}$, then Orbit D should lie entirely inside and outside the asteroid’s magnetosphere, respectively. For dipole moments between these two limits, we expect that the spacecraft will pass in and out of the magnetosphere during Orbit D. Equivalent but higher limiting moment values can be assigned for Orbits A, B, and C and the transfer orbits. Each of these three cases will be analyzed to obtain a moment estimate using a distinct approach.

Similarly, we can obtain a constraint on Psyche’s dipole moment by recasting [Eq. (1)] in terms of the subsolar magnetopause stand-off distance, $R_{\mathrm{MP}}$, at which the magnetic pressure due to the body is balanced by the solar wind pressure:

10 $$ M_{\mathrm{P}} \approx 4\pi v_{\mathrm{sw}} R_{\mathrm{MP}}^{3} \sqrt{\frac{2 \rho _{\mathrm{sw}}}{F\mu _{0}}} $$

This relation, as well as the aforementioned hybrid simulations, requires knowledge of the solar wind speed. Because the Psyche spacecraft does not carry a solar wind particle instrument, we can use solar wind velocity and IMF estimates from semi-empirical and physics-based solar wind propagation models that are constrained by measurements taken at 1 AU (Merkin et al. 2016; Odstrcil 2003; Oran et al. 2013).

The simplest situation will be if the spacecraft never passes through a magnetopause boundary even during Orbit D, such that $\vec{B}_{\mathrm{der}}$ is dominated by $\vec{B}_{\mathrm{der},\mathrm{e}}$. In this case, the analytical relationship [Eq. (10)] and our hybrid simulations (Fig. 7) indicate that $M_{\mathrm{P}} \lesssim 10^{13}~\text{Am}^{2}$.

Due to the variability of the solar wind and the body’s rotation, we expect the magnetospheric topology to significantly change over time. While large planetary magnetospheres have extended regions with a quasi-fixed topology, a small magnetosphere of a quickly rotating body would continuously change its configuration as it reconnects to the external fields. Because of the periodicity of the rotation and the fact that similar wind condition would repeat during the mission, we will collect data from various times and bin them as a function of spacecraft locations in the PBF and PSO frames. This should enable us to construct several separate mean configurations for further study. For orbits that pass into and out of the magnetosphere, we will use the binned data to map the magnetopause surface. Magnetopause crossings can be identified from observing abrupt changes in the angle of $\vec{B}_{\mathrm{der}}$ (e.g., by tens of degrees), increases in $B_{\mathrm{der}}$ (e.g., by factors of several or more), and/or changes in the several Hz frequency variations of $B_{\mathrm{der}}$ (e.g., by up 1–2 orders of magnitude) (Winslow et al. 2013). These indicators can be collected and used to determine the magnetopause boundary. These indicators can be collected and used to determine the magnetopause boundary to estimate $M_{\mathrm{P}}$ from Eq. (10).

Orbits for which the spacecraft spends a large fraction of the orbit inside the magnetosphere will yield $\vec{B}_{\mathrm{der}}$ that may be dominated by $\vec{B}_{\mathrm{der},\mathrm{i}} $. Such measurements will enable a first order estimate of dipole moment magnitude by fitting the data to a simple dipole approximation analogous to Eq. (4):

11 $$ \vec{B}_{\mathrm{der}} ( \vec{r}_{p} ) = \frac{\mu _{0}}{4\pi} \biggl[ \frac{3 \vec{r} ( \vec{M}_{\mathrm{P}} \boldsymbol{\cdot} \vec{r}_{p} )}{r_{P}^{5}} - \frac{\vec{M}_{\mathrm{P}}}{r_{P}^{3}} \biggr] $$

Although the magnetosphere is compressed by the wind and deviates from a pure dipole, this effect is most prominent at the outer regions of the magnetosphere, while closer to the body the field becomes increasingly dipolar (e.g., within $2~R_{\mathrm{P}}$ for body with dipole moment $>10^{15}~\text{Am}^{2}$; Fig. 7F).

We can use the above estimates of the moment to model the solar wind interaction with the asteroid using hybrid simulations (e.g., Fig. 7) to obtain a measure of how the wind distorts the field away from the dipole field in Eq. (11). Such a simulation will solve for the magnetospheric currents and induced currents self-consistently. We can then subtract the simulated external field from the data, and thus remove the contribution of the Sun-tied, time dependent external field to yield a map of Psyche’s internal field,

$$ \vec{B}_{\mathrm{der},\mathrm{i}} '\approx \vec{B}_{\mathrm{der}} - \vec{B}_{\mathrm{der},\mathrm{e}} ' $$

This procedure would be done iteratively to yield a more accurate estimate of $\vec{M}_{\mathrm{P}}$.

#### Magnetic Anomaly Map and Higher-Order Gauss Coefficients

Although only an estimate of the dipole moment is required for the level 1 requirement, spatial maps of the remanent magnetic field of Psyche (i.e., a magnetic anomaly map) and associated higher order Gauss coefficients would provide bonus science. As discussed below, the Gauss coefficient power spectrum could constrain the depth of the magnetization and its horizontal spatial scale. Combined with anomaly maps, the magnetic power spectrum could enable a correlation between the NRM and possible geologic terranes (e.g., craters, volcanic edifices, tectonic features, and/or endogenous and exogenous surface materials with varying compositions and mineralogies). This could in turn constrain the age and source of the NRM (internal versus external fields) and in turn constrain the thermal and magnetic evolution of the asteroid. For example, the demonstration that Psyche’s magnetization was unidirectional or not could be used to distinguish between external field sources like the nebular field and internal field sources like a dynamo, assuming scrambling of the magnetization by impact-induced block rotation can be excluded (Sect. 1.2).

Obtaining a magnetic anomaly map would require that Psyche’s dipole moment was sufficiently large that the spacecraft spends a large fraction of time in a magnetosphere (e.g., at or below 170 km for a Psyche moment at the level 1 value). In this case, a map could be obtained of $\vec{B}_{\mathrm{der},\mathrm{i}} '$ as a function of radial coordinates $r$, $\theta _{P}$ and $\phi _{P}$. This map could be downward- or upward-continued to a fixed altitude $r_{0}$ to yield a magnetic anomaly map,

$$ \vec{B}_{\mathrm{rf},\mathrm{anom}} ( r_{0}, \theta _{P}, \phi _{P} ) = \vec{B}_{\mathrm{der},\mathrm{i}} '( r_{0}, \theta _{P}, \phi _{P} ). $$

If this map has sufficiently complete coverage in $\theta _{P} $ and $\phi _{P}$, we can use least squares fitting to obtain the higher order Gauss coefficients:

12 $$ \vec{B}_{\mathrm{rf},\mathrm{anom}} ( r_{0}, \theta _{P}, \phi _{P} ) =- \nabla \sum_{l=1}^{N_{\mathrm{i}}} \sum_{m=0}^{l} r_{0} \biggl( \frac{r_{0}}{r_{P}} \biggr)^{l+1} P_{l}^{m} ( \cos \theta _{P} ) \bigl( g_{l}^{m, \mathrm{i}} \cos m \phi _{P} + h_{l}^{m, \mathrm{i}} \sin m \phi _{P} \bigr) $$

where $l$ and $m$ are the degree and order (integers), $g_{l}^{m,\mathrm{i}}$ and $h_{l}^{m, \mathrm{i}}$ are the internal Gauss coefficients, $N_{\mathrm{i}}$ is an integer specifying the highest orders in the internal and external fields, $P_{l}^{m}$ are the Schmidt semi-normalized associated Legendre polynomials (Blakely 1996). Note that the dipole moment estimate (Sect. 4.6.2) can be expressed as:

$$ M_{\mathrm{P}} = \bigl( {4\pi r_{0}^{3}} / {\mu _{0}} \bigr) \sqrt{ \bigl( g_{1}^{0, \mathrm{i}} \bigr)^{2} + \bigl( h_{1}^{0, \mathrm{i}} \bigr)^{2} + \bigl( h_{1}^{1, \mathrm{i}} \bigr)^{2}} $$

As dicussed below, the Gauss coefficients provide information about the angular spatial scale and depth of the magnetization distribution with the body.

The power of the magnetic field is defined as the mean-squared average of the magnetic-field intensity at a given radius $r$:

13 $$ R_{l} = \biggl( \frac{a}{r} \biggr)^{2l+4} ( l+1 ) \sum_{m=0}^{\boldsymbol{l}} \sqrt{ \bigl( g_{l}^{m,i} \bigr)^{2} + \bigl( h_{l}^{m,i} \bigr)^{2}} $$

This quantity, and how it varies as a function of $l$, can be computed easily when the Gauss coefficients are known (Lowes 1966). To assess how the strength of the magnetic field might vary beyond the simple dipole term, we make use of a stochastic magnetization model (Voorhies et al. 2002; Wieczorek 2018). The model considered here consists of an ensemble of magnetized thin spherical caps that are confined to lie between two depths below the 111 km mean radius of the body. Each spherical cap is set to have the same angular radius and is assumed to possess a random magnetization intensity, a random direction of magnetization and a random location. After averaging over the random variables, a simple analytic equation can be shown to exist for the power spectrum.

The number of spherical caps and their mean-squared magnetization are part of a factor that multiplies the theoretical power spectra and that does not otherwise affect the power spectrum shape (Wieczorek 2018). Furthermore, Wieczorek (2018) demonstrated that the depth of the bottom of the magnetized zone, $d_{b}$, has only a small impact on the spectra, especially at the low spherical-harmonic degrees that will be resolved by the Psyche mission. Wieczorek (2018) also demonstrated that different assumptions about the directions of magnetization would affect the magnitude of the power spectrum by no more than a factor of about two. The two parameters that have the largest effect on the shape of the spectra are the radii of the magnetized disks, $\theta _{0}$, and the depth to the top of the magnetized region, $d_{t}$.

**Fig. 13:**
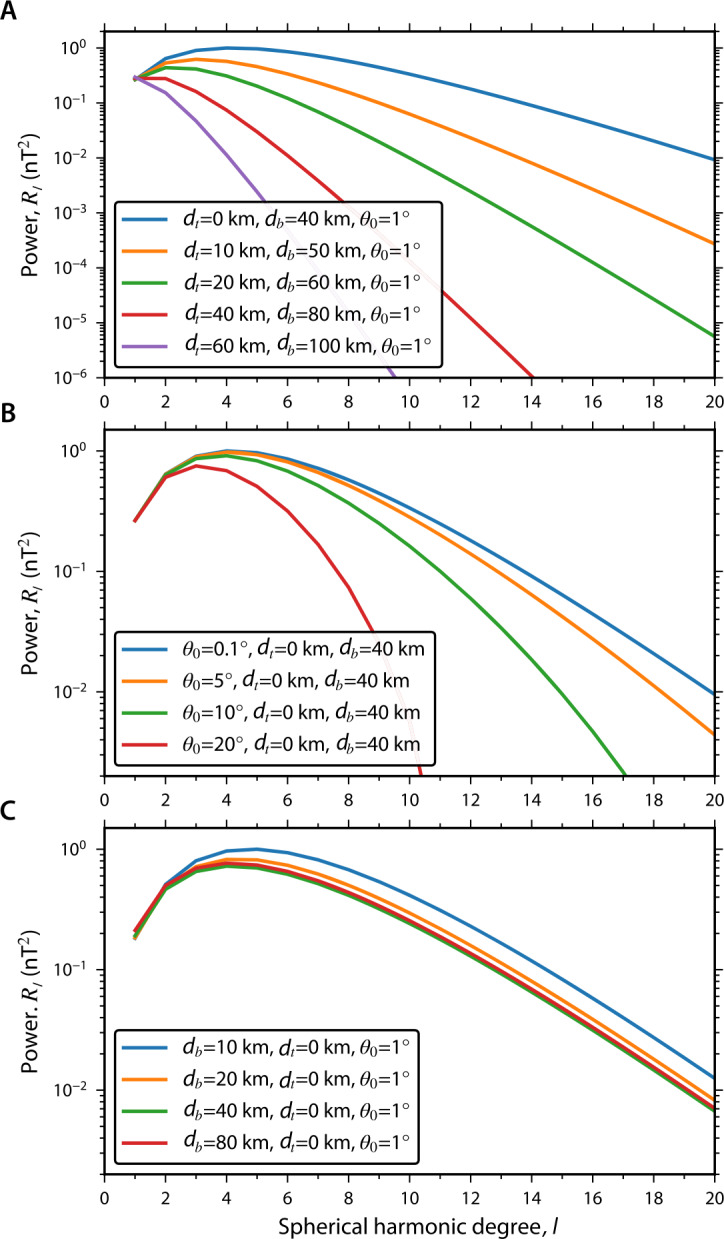
Magnetic field power spectra predicted by a stochastic magnetization model. The magnetization consists of thin spherical caps that have random magnetization directions and intensities. The magnetized spherical caps are defined by their angular radius, $\theta _{0}$, and reside within an annulus that is defined by the depths to the top and bottom of the magnetized zone, $d_{t}$ and $d_{b}$, respectively. The power spectra are computed at a radius of 145 km and it is assumed that Psyche can be approximated by a sphere with a mean radius of 113 km. (**A**) Dependence of the power spectra on the depth to the top of the magnetized region beneath the surface, which varies from 0 to 60 km. (**B**) Dependence of the power spectra on the angular radius of the individual magnetized spherical caps, which varies from 0.1 to $20^{\circ}$. (**C**) Dependence of the power spectra on the depth to the bottom of the magnetized region beneath the surface, which varies from 10 to 80 km

To understand the dependence of the Gauss coefficients on magnetization depth and spatial scale, we calculated three sets of magnetic power spectra. First, we set the angular radius of the spherical caps to $1^{\circ}$ and varied the depth to the top of the magnetized region from the surface to 60 km (Fig. 13A). We fixed the thickness of the magnetized zone to be equal to the 40-km coherence scale discussed in Sect. 1.3. For spherical-harmonic degrees near 10, the magnitudes of the spectra vary by about 6 orders of magnitude. Next, we placed the top of the magnetized zone at the surface and the bottom depth at 40 km, and calculated spectra where the angular radii of the spherical caps vary from 0.1 to $20^{\circ}$ (corresponding to diameters of about 0.5 to 80 km at the surface) (Fig. 13B). Near degree 10, this parameter is seen to affect the magnitude of the power spectra by about 2 orders of magnitude. In particular, these models show that the maximum power occurs near degrees 3–4 and that the power for these degrees can be 4–5 times larger than the corresponding power of the dipole term. Finally, we again placed the top of the magnetized zone at the surface and varied the bottom depth from 10 to 80 km (Fig. 13C). The latter affirms the lack of sensitivity of the spectra to bottom depth previously observed by Wieczorek (2018).

These example spectra show that if the Gauss coefficients of Psyche can be determined for degrees beyond the dipole term, that it should be possible to constrain the size and depth of the magnetic sources. We emphasize that the theoretical power spectra of Wieczorek (2018) were developed for planetary bodies that could be approximated as being spherical. Although we do not expect the nonspherical shape of Psyche to affect the broad conclusions taken from Fig. 13, more complex magnetization models that consider the real shape of Psyche will need to be investigated.

### Archiving

Data products will be archived as daily Common Data Format (CDF) files with Planetary Data Systems (PDS) Small Bodies Node using the PDS4 standards. Data products at the Raw, Partially Processed and Calibrated levels will be archived continuously throughout the mission as the data are received from the spacecraft and processed through the Magnetometer data pipeline. Data products up to the Calibrated data level will have a measurement cadence of $f_{s} = 50$ Hz. The Derived products will be archived continuously during orbital operations with a measurement cadence of 1 Hz and the Remanent Field products will be archived at the end of the primary mission.

## In-Flight Calibration

### Overview

The pre-flight calibration campaign focused on determining the scale factors, orthogonality and offsets, of the SUs and EUs (Sect. 3.5). However, because this campaign was conducted before the Magnetometer was mounted on the spacecraft did not quantify noise from the flight system. To address the latter, there will be three additional in-flight campaigns: an initial checkout campaign, solar wind monitoring, and solar panel rotation. These will give regular estimates of the offsets and flight system field during cruise and while in orbit around the asteroid.

### Initial Checkout

During initial checkout activities occurring just after launch, the Magnetometer will be turned on and passively measure the flight system magnetic field as other subsystems are progressively turned on and cycled through their various modes and power states. The data collected during initial checkout will be used to build calibration tables that include both the expected field contribution from each subsystem as well as coupling coefficients based on the changes in the observed field resulting from the subsystems. These tables will later be updated using information collected throughout cruise and during a flyby of Mars. During orbital operations, the calibration table will be used along with ancillary data indicating what subsystems are being commanded to inform the removal of flight system generated fields using gradiometry and frequency filtering of the differenced field (Sect. 4.4).

### Solar Wind Monitoring

In addition to the passive monitoring of the flight system components, there will be a dedicated solar wind monitoring interval during initial checkout for determination of the zero-level field, $\vec{B}_{\mathrm{zl}}$. Correcting for the zero-level field is part of the Calibrated data processing step (Sect. 4.4). Solar wind monitoring takes advantage of the Alfvénic nature of the IMF (Belcher 1973; Davis and Smith 1968) for which the ratio of the magnitude of the compressional fluctuations to the transverse fluctuations is low. This means that when there are changes in the direction of the IMF, its magnitude stays relatively constant. However, when a non-rotating spacecraft field is added to the IMF, the magnitude of the total observed field will change. The zero levels, which include both the flight system DC field and the residual instrument zero level offsets, are given by the corrections to the magnetic field components that keep the magnitude of the field fixed during the observed IMF rotations (Leinweber et al. 2008).

In particular, time sequences of the ambient field can be used to estimate the zero-level field for each SU-EU pair as those values that minimize the variance of the squared magnitude of the measured field $\vert \vec{B}_{\mathrm{cal},\mathrm{SA},\mathrm{FDF},\mathrm{SC}, n} \vert ^{2}$ where $\vec{B}_{\mathrm{cal},\mathrm{SA},\mathrm{FDF},\mathrm{SC}, n}$ data have been already cleaned of $10^{-5}$ to 0.1 Hz, >0.1 Hz, and step changes (note that other intermediate Calibrated data products like $\vec{B}_{\mathrm{cal},\mathrm{FDF},\mathrm{SC}, n}$ could be used instead here) (Leinweber et al. 2008). This is equivalent to solving the system of linear equations for $\vec{B}_{\mathrm{zl}}$:

14 $$ \boldsymbol{A}_{\boldsymbol{n}} \vec{B}_{\mathrm{zl}, n} = \vec{b}_{n} $$

Here, the covariance matrix

$$ \boldsymbol{A}_{\boldsymbol{n}} = \left [ \textstyle\begin{array}{c@{\quad}c@{\quad}c} \langle B_{x}^{2} \rangle - \langle B_{x} \rangle ^{2} & \langle B_{x} B_{y} \rangle - \langle B_{x} \rangle \langle B_{y} \rangle & \langle B_{x} B_{z} \rangle - \langle B_{x} \rangle \langle B_{z} \rangle \\ \langle B_{y} B_{x} \rangle - \langle B_{y} \rangle \langle B_{x} \rangle & \langle B_{y}^{2} \rangle - \langle B_{y} \rangle ^{2} & \langle B_{y} B_{z} \rangle - \langle B_{y} \rangle \langle B_{z} \rangle \\ \langle B_{z} B_{x} \rangle - \langle B_{z} \rangle \langle B_{x} \rangle & \langle B_{z} B_{y} \rangle - \langle B_{z} \rangle \langle B_{y} \rangle & \langle B_{z}^{2} \rangle - \langle B_{z} \rangle ^{2} \end{array}\displaystyle \right ] $$

where $B_{x}$, $B_{y}$, $B_{z}$ are the Cartesian components of $\vec{B}_{\mathrm{cal},\mathrm{SA},\mathrm{FDF},\mathrm{SC}, n}$, the angle brackets denote temporal means and

$$ \vec{b} \equiv \frac{1}{2} \left [ \textstyle\begin{array}{c} \langle B_{x} \vert \vec{B}_{\mathrm{cal},\mathrm{SA},\mathrm{FDF},\mathrm{SC}, n} \vert ^{2} \rangle - \langle B_{x} \rangle \langle \vert \vec{B}_{\mathrm{cal},\mathrm{SA},\mathrm{FDF},\mathrm{SC}, n} \vert ^{2} \rangle \\ \langle B_{y} \vert \vec{B}_{\mathrm{cal},\mathrm{SA},\mathrm{FDF},\mathrm{SC}, n} \vert ^{2} \rangle - \langle B_{y} \rangle \langle \vert \vec{B}_{\mathrm{cal},\mathrm{SA},\mathrm{FDF},\mathrm{SC}, n} \vert ^{2} \rangle \\ \langle B_{z} \vert \vec{B}_{\mathrm{cal},\mathrm{SA},\mathrm{FDF},\mathrm{SC}, n} \vert ^{2} \rangle - \langle B_{z} \rangle \langle \vert \vec{B}_{\mathrm{cal},\mathrm{SA},\mathrm{FDF},\mathrm{SC}, n} \vert ^{2} \rangle \end{array}\displaystyle \right ] $$

and where $\vert \vec{B}_{\mathrm{cal},\mathrm{SA},\mathrm{FDF},\mathrm{SC}, n} \vert ^{2} = B_{x}^{2} + B_{y}^{2} + B_{z}^{2}$.

### Solar Panel Rotation

The solar panels are expected to be the main subsystem contributing to the flight system field in the frequency range $10^{-5}$–$0.1~\text{Hz}$ due to their rotation about the spacecraft $y$ axis as they track the sun. This is a critical frequency range because it also contains the expected frequencies that will be observed at the asteroid as the spacecraft observes the remanent field while in orbit. An additional complication is that unlike most other flight system components with substantial magnetic fields, the solar arrays are larger in size than their minimum distance to SU2, such that their fields at SU2 will be highly nondipolar and therefore not easily removed with single dipole-fitting gradiometry techniques (Sect. 4.4). Furthermore, the solar arrays rotate about the FS $y$ axis, such that the fields at the SUs will depend on the solar array drive assembly (SADA) angle, $\theta _{S}$. Note that the solar panels are expected to always remain within $5^{\circ}$ of the Sun and to maintain an approximately (within 10%) constant current state during the full mission, such that the main source of field variation at the Magnetometer should be changes in the relative orientation of the SADA relative to the spacecraft bus rather than changes in currents.

We will therefore characterize the solar array-generated fields at each SU as a function of $\theta _{S}$. This will be accomplished by performing a series of calibration rolls where the spacecraft is rotated along the solar array axis for a duration of 8 hours over a total of 8 full $360^{\circ}$ rotations while keeping the solar arrays facing the sun. This activity will be repeated periodically throughout cruise and approach. The measurements collected by the Magnetometer during the rolls, $\vec{B}_{\mathrm{cal},\mathrm{FDF},\mathrm{SC}, n}$, will be used to compile a look-up table of the observed solar array field at each SU as a function of $\theta _{S}$. The value of $\theta _{S}$ at the start of the rolls is also recorded since the zero-level calibration depends on this. This table will then be used to subtract the solar array field from the Magnetometer data as part of producing Calibrated data from Partially Processed data (Sect. 4.4). These calibration rolls will be repeated periodically throughout cruise, during the Mars gravity assist and during approach to Psyche.

## Summary

The Psyche Magnetometry Investigation is designed to search for a remanent field around asteroid (16) Psyche that would provide evidence of past dynamo action. Evidence for a core dynamo would demonstrate that the asteroid was partially or fully differentiated rather than being an unmelted accretionary aggregate, thereby addressing one of the key mission science objectives. Using our knowledge of the age and intensity of NRM in meteorites and a nascent but growing understanding of dynamo generation on planetesimals, we conclude that a demonstration that Psyche has a moment of at least $2\times14~\text{Am}^{2}$, which would produce a field of up to $\sim6~\text{nT}$ at the lowest planned mapping altitude (75 km), would be consistent with a past core dynamo. However, given the wide range of NRM intensities in iron meteorites, the field at this altitude might range up $\sim10{,}000~\text{nT}$, which would make Psyche one of the most magnetic objects encountered by a spacecraft. As such, a key challenge was to design a Magnetometry Investigation with sufficient Reconstructed Measurement Uncertainty for detecting the minimum dipole moment while also having a high dynamic range to avoid saturation of the most strongly magnetized conceivable possibilities. The Psyche Magnetometry Investigation achieves this using high-heritage dual three-axis fluxgate sensors derived from the VFM on the Swarm mission, gradiometry combined with a stringent magnetic cleanliness program for the flight system, a series of pre-flight and in-flight calibration activities, and a ground data processing program that applies these calibrations, suppresses flight system fields, and constructs magnetic moment estimates from modeling of magnetic field data.
